# Simple simultaneous analysis of various cardiovascular drug mixtures with vincamine: comparative eco-friendly assessment

**DOI:** 10.1186/s13065-024-01303-2

**Published:** 2024-10-10

**Authors:** Sara S. Mourad, Magda A. Barary, Amira F. El-Yazbi

**Affiliations:** Pharmaceutical Analytical Chemistry Department, Faculty of Pharmacy, Alexandria University, 1 El Khartoum Square, Alexandria, 21521 Egypt

**Keywords:** Cardiovascular drugs, Vincamine, MEKC, HPLC, Greenness, Blueness, Whiteness

## Abstract

The development of two eco-friendly analytical methods for the simultaneous determination of eight cardiovascular drugs; hydrochlorothiazide (HCT), captopril (CPL), lisinopril (LSP), valsartan (VAL), atorvastatin (ATR), bisoprolol (BSL), amlodipine (AML) and carvedilol (CVL); alongside with the nutraceutical vincamine (VIC) is essential for sustainable pharmaceutical analysis. This study explores the application of Micellar Electro Kinetic Chromatography (MEKC) and High-Performance Liquid Chromatography (HPLC) for this purpose. In MEKC method, the separation was done using fused silica capillary (41.5 cm × 50 µm id) and a back ground electrolyte consisting of 50 mM borate buffer (pH 9) containing 50 mM sodium lauryl sulphate (SLS) and 10% organic modifier (Acetonitrile). In HPLC method, separation was performed on a ZORBAX Extend-C18 (4.6 × 250 mm, 5 µm) column, using a gradient mobile phase consisting of 50 mM phosphate buffer pH 3 and methanol. Both methods attained good linearity (r ≥ 0.9996) with low values of LOD and LOQ. Both methods were successfully applied in the determination of co-administered single, binary and ternary dosage form of the studied drugs. Moreover, application of various combinations of co-administered dosage forms was achieved in rat plasma, confirming the applicability of these methods in different matrices. The use of micellar solutions in MEKC enhances separation efficiency while reducing the need for organic solvents, aligning with green chemistry principles. HPLC methods were optimized using environmentally benign solvents, ensuring reduced toxicity and waste production. The methodologies were evaluated through green, white, and blue metrics to ensure comprehensive sustainability, considering ecological impact, safety, and practical efficiency. These methods were not only cost-effective and time-saving but achieved high efficiency, sensitivity, and reproducibility making them ideal for routine use in pharmaceutical analysis.

**Supplementary Information:**

The online version contains supplementary material available at 10.1186/s13065-024-01303-2.

## Introduction

Cardiovascular disorders (CVDs) continue to be the primary global cause of illness and mortality, accounting for 17.9 million deaths yearly, according to the World Health Organization (WHO) [1]. These disorders encompass a range of conditions affecting the heart and blood vessels, such as coronary artery disease, hypertension, heart failure, and arrhythmias. Effective management of these disorders is crucial for reducing the associated health burden and improving patient outcomes. Pharmacotherapy plays a pivotal role in the treatment of CVDs, with a diverse array of cardiovascular drugs employed to address the underlying pathophysiology, alleviate symptoms, and prevent complications [2].

Cardiovascular drugs, including antihypertensives, antianginals, anticoagulants, antiarrhythmics, lipid-lowering agents, and vasodilators, form the cornerstone of CVD management. These medications function through various mechanisms to help avoid blood clots, lower cholesterol, or lower blood pressure, regulate heart rhythms, and enhance cardiac output. Their targeted actions not only mitigate the immediate risks associated with cardiovascular events but also contribute to long-term cardiovascular health [3, 4].

**Fig. 1:**
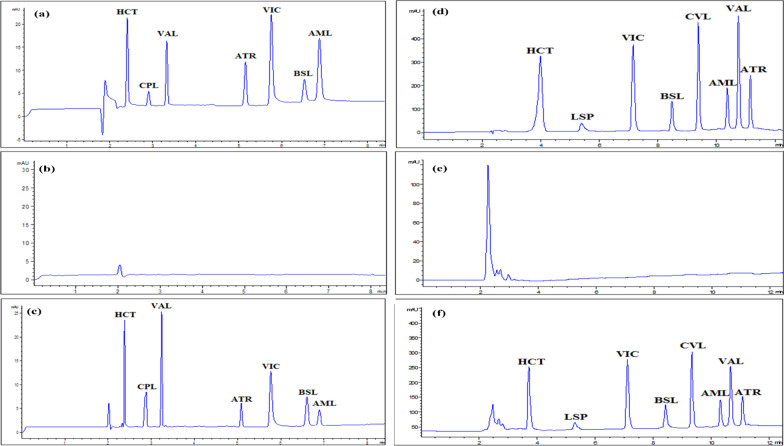
**a**–**c** Electropherograms (220 nm) of the proposed MEKC method showing **a** a standard solution containing equal ratio 50 μg/mL of HCT, CPL, VAL, ATR, VIC, BSL and AML, **b** blank rat plasma **c** 100 μL spiked rat plasma with 100 μg/mL HCT, ATR, VIC, BSL, AML, 200 μg/mL CPL and 300 μg/mL VAL. **d**–**f** Chromatograms (220 nm) of the proposed HPLC method showing **a** a standard solution containing equal ratio 50 μg/mL of HCT, LSP, VIC, BSL, CVL, AML, VAL and ATR, **b** blank rat plasma **c** 100 μL spiked rat plasma with 50 μg/mL of each drug

In this research, eight cardiovascular drugs from different classes were studied including, hydrochlorothiazide (HCT, Fig. 1a), which is the most common thiazide diuretic recommended for the treatment of hypertension and edema. captopril (CPL, Fig. 1b) and lisinopril (LSP, Fig. 1c) are angiotensin converting enzyme inhibitors (ACEIs) used to treat hypertension combined with beta blockers or thiazide diuretics. They are the only ACEIs that are not prodrugs. Valsartan (VAL, Fig. 1d) is one of the angiotensin II receptor blockers (ARBs) that preferentially attach to the angiotensin receptor 1 (AT1) and inhibit the binding of the angiotensin II protein, resulting in lowered blood pressure, decreased aldosterone levels, decreased cardiac activity, and elevated salt excretion. Atorvastatin (ATR, Fig. 1e) functions by competitively inhibiting the enzyme hydroxymethylglutaryl-coenzyme A (HMG-CoA) reductase, which is responsible for catalyzing the conversion of HMG-CoA to mevalonic acid. This results in lipid lowering effect for those patients with high risk of CVDs. Amlodipine (AML, Fig. 1f) is a calcium channel blocker used to treat hypertension and angina. Bisoprolol (BSL, Fig. 1g) and carvedilol (CVL, Fig. 1h) are non-selective and β-1 adrenergic antagonists, respectively. Both are used to treat hypertension and myocardial infarction [5].

In addition to conventional cardiovascular drugs, there is growing interest in the potential therapeutic benefits of naturally derived compounds, such as vincamine. Vincamine (VIC, Fig. 1i), an alkaloid extracted from the Vinca minor plant, is utilized as a nutraceutical for its potential benefits in managing cardiovascular diseases. Known for its vasodilatory properties, vincamine enhances blood flow, particularly in cerebral vessels, which can help improve cognitive function and reduce symptoms related to poor circulation [6]. It also exhibits antioxidant and anti-inflammatory effects [7], which contribute to cardiovascular health by protecting the heart and blood vessels from oxidative stress and inflammation, common factors in cardiovascular diseases. As a supplement, vincamine is promoted for its ability to support overall cardiovascular wellness. Recent studies suggest that vincamine may offer valuable adjunctive benefits in the treatment of cardiovascular disorders [8].

Many analytical techniques for the separation and quantification of such drugs, either alone or in conjunction with other cardiovascular drugs, in various matrices, bulk, dose forms, and biological fluids, were reported in the literature.

Among the recent reported HPLC and CE methods for the studied drugs, the determination of a mixture of HCT/CPL was performed using HPLC [9–11] and CZE [12]. Also, the determination of a mixture of HCT/LSP was performed by HPLC [13–19], CZE [12] and MEKC [20] methods. For HCT/VAL mixture, the recent reported methods includes HPLC [9, 11, 21–25] and CE [12]. For HCT/VAL/AML mixture, HPLC [26–35] and CZE [36] methods were applied. In addition to previous mixtures, HCT/BSL mixture has been reported to be determined by HPLC [37–43]. Also, for the analysis of HCT/CVL mixture, HPLC [11, 44–47] and MEKC [48] methods are reported. A mixture of AML/VAL has been separated and quantified using HPLC [49–56] and CE [57–59]. A mixture of AML/ATR has been analyzed using HPLC [60–71] and CE [72]. A reported HPLC [73] method showed the simultaneous analysis of AML/VAL/ATR together. For VIC, a nutraceutical of interest, various HPLC [74–76] methods were reported for its determination.

This paper aims to develop and validate robust analytical methods, Micellar Electro Kinetic Chromatography (MEKC) and High-Performance Liquid Chromatography (HPLC), for the simultaneous separation and quantification of multiple cardiovascular drugs, HCT, CPL, LSP, VAL, ATR, BSL, AML and CVL, together with vincamine, dietary supplement, in a single run. This study seeks to establish a comprehensive, reliable, and efficient analytical methods capable of supporting the simultaneous analysis of multiple dosage forms of cardiovascular drugs, including emerging natural compounds like vincamine in a green, blue and white perspectives.

## Experimental

### Instrumentation

Agilent CE equipment for data manipulation (Agilent Technologies, Waldbronn, Germany, series 7100) with DAD and a PC equipped with Agilent Chem Station software. The capillary that was utilized was acquired from Agilent Technologies, Inc. A deactivated fused silica capillary with an i.d. of 50 μm and a total length of 50 cm and an effective length of 41.5 cm was used.

Agilent 1200 series HPLC–DAD (vacuum degasser, auto-injector, quaternary pump, diode array, and multiple wavelength detectors G1315 C/D and G1365 C/D) was utilized. It was linked to a PC running Agilent ChemStation Software (Agilent Technologies, Santa Clara, CA, USA). The chromatographic separation was achieved using a ZORBAX Extend-C18 (4.6 × 250 mm, 5 µm) column (Agilent Technologies, Santa Clara, CA, USA).

A 4-digit German analytical balance, the Kern AEJ 220–4 M balance, was utilized to weigh drugs and dosage forms precisely, while a 3-digit Sartorius BL 310 balance was utilized for accurate weighing of buffer and surfactant. Jenway model 3505 (Germany) pH meter was utilized for pH adjustment throughout the study. Christ rotational vacuum concentrator, Germany was used in plasma sample extraction.

### Materials and reagents

HCT (purity > 99.5%, Pharco Pharmaceuticals Co., Alexandria, Egypt), CPL (purity > 99.5%, EIPICO pharma, Egypt), VAL (purity 99.7%, Novartis Pharma S.A.E., Cairo, Egypt), ATR (purity 99.8%, EIPICO pharma, Egypt), VIC (purity 99%, October pharma, Egypt), BSL (purity > 99%, Global Napi Pharmaceuticals, 6th October, Egypt), CVL (purity > 99.8%, Chemipharm Pharmaceutical Industries, Egypt), LSP (purity > 99.85%, AstraZeneca, Egypt) and AML (purity 99.8%, Pfizer Egypt S.A.E., Cairo, Egypt) were used in this study.

All dosage forms utilized in the study were purchased from the local market. Capoten 50® (50 mg CPL/tablet), Zestril 10® (10 mg LSP/tablet) Tareg 80® (80 mg VAL/tablet), Ator 10® (10 mg ATR/tablet), Concor 10® (10 mg BSL/tablet), Norvasc 10® (10 mg AML/tablet), Dilatrol 25® (25 mg CVL/tablet), and Brain Ox® (30 mg VIC/capsule. Co-tareg® (80 mg VAL and 12.5 mg HCT/tablet), Exforge ® (10 mg AML and 160 mg VAL/tablet), Exforge HCT® (10 mg AML,160 mg VAL and 12.5 mg HCT/tablet), Capozide® (50 mg CPL and 25 mg HCT/tablet), Zestoretic® (20 mg LSP and 12.5 mg HCT), Concor plus® (10 mg BSL and 25 mg HCT/tablet), Caduet® (5 mg AML and 10 mg ATR/tablet) and Co-dilatrol® (25 mg CVL and 12.5 mg HCT). In addition, HCT laboratory made tablets labeled to contain 25 mg HCT due to the lack of its commercial dosage form were used.

Boric acid (Oxford Lab Chem, Mumbai, India), HPLC grade ethyl acetate and orthophosphoric acid (LAB-SCAN Analytical Sciences, Poland), HPLC grade acetonitrile and methanol (Sigma-aldrich Chemie GmbH, Buchs, Switzerland), sodium hydroxide and sodium lauryl sulphate (SLS) and phosphate monobasic (El-Nasr Chemical Industry company, Egypt) and deionized water were used.

### Animals

The study utilized adult male Wistar rats weighing 200–250 g from the Faculty of Pharmacy animal facility at Alexandria University in Alexandria, Egypt. The Institutional Animal Care and Use Committee at Alexandria University in Egypt granted approval for all experimental procedures and animal manipulations (Approval No. AU/06.2023.4.12.2.147).

After the rats were given isoflurane anesthesia, about 2 mL of blood samples were taken from their orbital sinus (retro-orbital plexus). Blood was collected in precoated tubes coated with ethylene diamine tetra-acetic acid after the retro-orbital venous plexus was punctured using un-heparinized glass capillary tubes. The tubes were then centrifuged at 1200 rpm for 15 min. For additional examination, the supernatant plasma layer was collected into Eppendorf tubes and kept at – 80 ℃. Lastly, rats were euthanized by overdose of thiopental (100 mg/kg).

### Experimental steps and calibration graphs construction

#### MEKC running buffer and HPLC mobile phase preparation

In MEKC, deionized water was used to prepare 50 mM borate buffer, which was then adjusted to pH 9 with 0.5 M NaOH in a 100 mL volumetric flask. The previously made buffer was used to prepare a 50 mM SLS. The finally used back ground electrolyte (BGE) consists of 50 mM borate buffer (pH 9) containing 50 mM SLS and HPLC-grade acetonitrile (90:10, v/v), respectively.

In HPLC, 50 mM phosphate buffer pH 3 and methanol were used in a gradient elution starting with the ratio 70:30 by volume. This ratio was changed at 5, 7, 8 min, where methanol was increased to be 60, 80 and 90%, respectively. The methanol ratio was restored to 30% before subsequent injections.

#### MEKC procedure

The daily conditioning of the capillary was set that the capillary was flushed by 0.5 M NaOH for 10 min, then water for another 10 min. Afterwards, 0.1 M NaOH for 5 min, waiting 2.5 min to ensure full activation of the inner wall of the capillary, then washed with water for 5 min. Lastly, it was allowed to equilibrate with BGE for 10 min.

To maintain proper repeatability of run-to-run injections, buffer vials were replenished after every 5 consecutive runs. Between successive runs, the capillary was washed for 2 min with the BGE.

Using the hydrodynamic mode, injections were made at the anodic side for 10 s at a pressure of 50 mbar. The applied voltage was constant at 30 kV. The analysis was performed at wavelengths 210 and 220 nm.

#### Calibration graphs: stock and working solution preparation

Standard stock solutions of 2000 μg/mL of HCT, CPL, LSP, VAL, ATR, VIC, BSL, AML and CVL were prepared in HPLC-grade methanol. For MEKC, working solutions were prepared using proper aliquots of the stock solutions to cover the concentration ranges of 5–50 μg/mL for HCT, ATR, BSL and AML, 10–100 μg/mL for CPL and VIC and of 20–200 μg/mL for VAL. Similarly, in HPLC method, the concentration ranges were 0.5–50 μg/mL for HCT, VIC, CVL and ATR, 1–50 μg/mL for LSP, BSL and AML and of 2–200 μg/mL for VAL (Table 2). The final dilution was performed with distilled water or methanol in MEKC or HPLC, respectively. For every solution, three injections were made.

#### Analysis of pharmaceutical preparations

##### Assay of single dosage forms

Ten tablets of each single dosage forms (Capoten 50®, Tareg 80®, Ator 10®, Concor 10®, Zestril 10®, Dilatrol 10® and Norvasc 10®) were weighed and finely grounded powder. A set of 25 mL volumetric flasks were filled with precisely weighed quantities of each powdered tablet, and the mixture was sonicated for 15 min in 15 mL methanol. After the flasks were completed to final volume, stock solutions containing 2 mg/mL were obtained by filtration (Whatman filter paper, Grade 1, 110 mm). Similar procedure was performed for HCT in its laboratory made tablets (containing 25 mg HCT/tablet as in the brand Hydrex®, together with standard tablet excipients). For BrainOX® capsules, the content of 10 capsules were taken and an accurately weighed portion of VIC was treated similarly. Serial dilutions of each extracted single dosage forms were prepared to the required concentrations (Table S3). For every solution, three injections were made.

##### Assay of binary and ternary dosage forms

Ten tablets of each combined dosage form were weighed and finely powdered. An accurately weighed portions of Co-tareg® (80 mg VAL/12.5 mg HCT) or of Exforge® (10 mg AML/160 mg VAL) or of Exforge HCT® (10 mg AML/160 mg VAL/12.5 mg HCT) or of Capozide® (50 mg CPL/25 mg HCT) or of Concor plus® (10 mg BSL/ 25 mg HCT) or of Caduet® (5 mg AML/10 mg ATR) or of Zestoretic® (20 mg LSP and 12.5 mg HCT) or of Co-dilatrol® (25 mg CVL and 12.5 mg HCT) were put into a series of 25 mL volumetric flasks and sonicated in 15 mL of methanol for 15 min. After the flasks were completed to final volume, stock solutions containing 2 mg/mL were obtained by filtration (Whatman filter paper, Grade 1, 110 mm). Serial dilutions of each extracted dosage form were prepared to the required concentrations (Table 3). For every solution, three injections were made.

#### Plasma sample preparation

Using the protein precipitation method, the studied drugs were recovered from spiked rat plasma. A centrifuge tube was filled with an aliquot of 100 μL of blank rat plasma that had been spiked with various aliquots of the drugs under study. Next, 2 mL of ethyl acetate was added. The tubes were then centrifuged for 15 min at 6000 rpm after being vortexed for 5 min. In a vacuum concentrator set at 40 ℃, the supernatant was separated and evaporated until it was completely dry and the residue was reconstituted with 1 mL water or 100 μL methanol to be injected after filtration (using 0.22 μm Millipore filter) in the MEKC or HPLC system, respectively.

#### Calibration in plasma and quality control standards

Spiking 100 μL of blank rat plasma with different aliquots of the prepared working standard solutions for the studied drugs followed by extraction, evaporation and reconstitution as illustrated in Sect. “Plasma sample preparation”. to get final concentration ranges of 50–1000 μg/mL for HCT, ATR, BSL and AML, 100–1000 μg/mL for CPL, 10–1000 μg/mL for VIC and 50–2000 μg/mL for VAL for MEKC system. Similarly, in HPLC system, the final concentration ranges reached were 1–100 μg/mL for HCT, LSP and AML, 2–100 μg/mL for VIC, 5–100 μg/mL for BSL, CVL and ATR and 2–200 for VAL (Table 4). As shown in tables S6 & S7, the four reported quality control (QC) samples were prepared similarly to the calibration standards in order to ensure precision and accuracy. They were then handled to obtain the final concentrations for the LLOQ (lower limit of quantitation), LQC (low quality control), MQC (medium quality control), and HQC (high quality control) for each analyte.

#### Determination of different co-administered dosage forms of the studied drugs with vincamine in spiked plasma samples

Cardiovascular diseases require multiple therapy that results in co-administration of different dosage forms. Brain OX® (VIC) is a nutraceutical that is commonly administered in most of cardiovascular diseases. Thus, the determination of various co-administered cardiovascular drugs together with VIC in rat plasma was performed using MEKC and HPLC methods.

Volumes of 100 μL blank rat plasma were spiked with different aliquots of drugs in the ratio of the co-administered dosage forms in different combinations as in illustrated in Table 5. The samples were treated as previously mentioned regarding the extraction, evaporation, reconstitution and injection.

## Results and discussion

Two simple, rapid and selective methods, MEKC and HPLC, were suggested for the separation and simultaneous determination of various drugs commonly co-administered in different combinations for the treatment of cardiovascular diseases. Method I, involves the use of MEKC for the simultaneous separation of HCT, CPL, VAL, ATR, VIC, BSL and AML, whereas method II presents an HPLC method for the separation of HCT, LSP, VIC, BSL, CVL, AML, VAL and ATR. The multiple wavelength detector in both methods was efficiently applied for the quantification of each analyte at its optimum wavelength.

### Analysis conditions optimization

#### MEKC method

To determine the ideal conditions for the separation of the studied drugs in CE, several trials were conducted. Firstly, capillary zone electrophoresis (CZE) mode with various buffers were tried, as acetate buffer (10, 20, 50, and 100 mM) of pH 4.7, phosphate buffer (10, 20, 50, and 100 mM) of pH 7.4, and borate buffer (10, 20, 50, and 100 mM) of pH 9 were among the buffers used in the initial mode trials. Additionally, 50 mM borate buffer was tested at various pH levels. All of the previously mentioned CZE trials could not effectively separate the studied drugs as some drugs appeared at the same migration time. Also, VIC was eluted with the electro-osmotic flow (EOF) and was not well separated.

Secondly, the MEKC mode was accessed by addition of SLS at a concentration greater than its critical micelle concentration (CMC). Phosphate buffer (20 or 50 mM) with (25 or 50 mM SLS), and borate buffer of 50 mM with (25 or 50 mM SLS) were tried. Borate buffer with 50 mM SLS was able to effectively separate all drugs and VIC was eluted away from EOF. Unfortunately, the peak shapes showed some tailing and fronting, in addition to, some forked peaks.

Lastly, the addition of an organic solvent to the BGE was examined as a means of modifying the MEKC mode. It has been discovered that organic modifiers in MEKC have a variety of impacts. When organic solvent is added, the BGE velocity significantly decreases. Furthermore, because the organic modifier's viscosity and dielectric constant vary as its volume increases, the BGE velocity decreases even more. Adding an organic modifier to the MEKC electrolyte changes the silica surface and improves the capillary inner wall's wetting. This results in alterations to the zeta-potential and, ultimately, the EOF [77]. In conclusion, the use of acetonitrile, an organic solvent, in MEKC mode allowed the effective separation of the analyzed drugs (Fig. 1a).

Numerous factors influence the modified MEKC method of analyte separation. A number of parameters were examined, including buffer type and pH, buffer concentration, SLS concentration, the kind and amount of organic modifier, applied voltage, injection period, detecting wavelength and operating temperature.

By studying the pKa of the studied drugs (Table 1), the buffer pH was tried within the range of 7 to 11 with borate buffer concentration of 50 mM in CZE and it did not improve separation of the tested drugs. However, the pH shift had no effect on the drugs' separation order or resolution when MEKC and an organic modifier were added. The findings of comparing 50 mM borate buffer pH 9 with 50 mM phosphate buffer pH 7.4 revealed that the borate buffer has a shorter migration time, better baseline and peak shapes, and is more reproducible than the phosphate buffer. The total run time using borate buffer was 7 min, unlike in case of phosphate buffer, it exceeded 10 min. Furthermore, BSL and AML were not well-separated, with poor resolution with phosphate buffer (pH 7.4). Therefore, the buffer of choice was borate buffer with pH 9.

**Table 1:** Structure and systematic names of the studied drugs in the proposed MEKC and HPLC methods

Name	Structure	IUPAC name	pKa
a. Hydrochlorothiazide (HCT)		6-chloro-1, 1-dioxo-3, 4-dihydro-2H-1, 2, 4-benzothiadiazine-7-sulfonamide	9.09
b. Captopril (CPL)		(2S)-1-[(2S)-2-methyl-3-sulfanylpropanoyl] pyrrolidine-2-carboxylic acid	4.02
c. Lisinopril (LSP)		(2S)-1-[(2S)-6-amino-2-{[(1S)-1-carboxy-3-phenylpropyl]aminohexanoyl] pyrrolidine-2-carboxylic acid	3.17, 10.21
d. Valsartan (VAL)		(2S)-3-methyl-2-[pentanoyl-[[4-[2-(2H-tetrazol-5yl) phenyl] phenyl] methyl] amino] butanoic acid	4.35
e. Atorvastatin (ATR)		(3R,5R)-7-[2-(4-Fluorophenyl)-3-phenyl-4-(phenylcarbamoyl)-5-propan-2-ylpyrrol-1yl]-3,5-dihydroxyheptanoic acid	4.31
f. Amlodipine (AML)		3-ethyl 5-methyl 2-[(2-aminoethoxy)methyl]-4-(2-chlorophenyl)-6-methyl-1,4-dihydropyridine-3,5-dicarboxylate	9.45
g. Bisoprolol (BSL)		1-[(propan-2-yl)amino]-3-(4-{[2-(propan-2-yloxy)ethoxy]methyl}phenoxy)propan-2-ol	9.67
h. Carvedilol (CVL)		1-(9H-carbazol-4-yloxy)-3-{[2-(2-methoxyphenoxy)ethyl]amino}propan-2-ol	8.74
i. Vincamine (VIC)		methyl (15S,17S,19S)-15-ethyl-17-hydroxy-1,11-diazapentacyclo[9.6.2.0^2^,⁷.0⁸^,1^⁸.0^15,1^⁹]nonadeca-2,4,6,8(18)-tetraene-17-carboxylate	6.7, 10.52

Borate buffer at pH 9 was used in various concentrations (10, 20, and 50 mM) to investigate the impact of buffer concentration. The data showed that low concentration buffer was uncapable of separating the seven drugs where some drug peaks were overlapped. The migration time of the analyzed drugs are influenced dramatically by increasing buffer concentration, resulting in longer separation periods and well-resolved and separated peaks. Finally, 50 mM concentration of borate buffer was selected to achieve better analysis with good resolution and peak shape in a reasonable migration time.

SLS concentrations of 15, 25, and 50 mM were added to the BGE along with 10% acetonitrile to examine the impact of SLS concentration on the separation. Both migration times and resolution increase with increasing SLS concentration. However, 15 mM SLS gave significantly tailed peaks, while 25 mM of SLS showed few better separations. 50 mM of SLS was the optimum concentration showing better peak shape in short analysis time.

The type and concentration of organic modifiers greatly influence how the drugs under study are separated. In the absence of organic modifier, both AML and BIS peaks were not well-separated with poor resolution. VIC peak was forked. Upon using 10% Acetonitrile, BSL and AML separation was significantly altered, showing better resolution. In addition, VIC was eluted with sharper peak. However, results were not repeatable when ≥ 20% (v/v) was utilized, as the high concentration of organic modifier can prevent micelle formation. Micelles are generally known to be unstable in water and organic solvent mixes that contain more than 20–30% organic solvent [77]. The peak shapes of both BIS and AML were deformed when methanol was used instead of acetonitrile. Moreover, ethanol was tried showing no significant change in the shape of the peaks. Also, it failed to improve the separation of the peaks of drugs with poor resolution. By increasing the percentage of ethanol, destabilization of micelles occurred. Therefore 10% (v/v) of acetonitrile was selected.

Different trials for applied voltage (20, 25 and 30 kV) utilizing the improved BGE are shown in Fig. S1. As expected, migration times increased with lowering voltage because of a drop in EOF, as seen in Fig. S1. In addition to increasing migration times, resolution was affected for VIC, BSL and AML. Therefore, a voltage of 30 kV was selected.

The pressure values of 20, 30, and 50 mbar did not significantly alter the migration time of the studied drugs. Due to its better response, 50 mbar was determined to be the ideal pressure. Peak width and height in hydrodynamic injection are influenced by injection time. Peak height and injection time are directly correlated under ideal circumstances. Sample solutions were injected at 50 mbar for 5 to 25 s, adjusting the injection time to find the ideal duration. Peak height increased with longer injection times; however, longer injection times also result in deformed peaks and a departure from linearity. Furthermore, altering the injection time did not result in any changes to the migration times of any of the analyzed drugs. Because of the good peak symmetry and linear relationship between peak height and injection volume, 10 s was determined to be the ideal injection time.

Fig. S4, shows UV spectra of the drugs to be studied in MEKC method. The proposed method permits separation of all drugs at 220 nm in ~ 7 min as shown in the obtained MEKC electropherogram (Fig. 1a). The quantitative determination of CPL, VAL, ATR and AML was done at 210 nm, whereas HCT, VIC and BSL was done at 220 nm. Moreover, DAD confirms peak purity as the purity angles were below the threshold values as illustrated in Fig. S4.

Within the FDA requirements, the suggested technique was found to have acceptable system suitability parameters: k’ > 2, N > 2000, α > 1, Rs > 2, and T ≤ 2 (Table S4) [3]. The peaks showed good resolution, symmetry, sharpness, and a suitable migration time.

The impact of varying the operating temperature (20, 25, and 30 °C) on the separation of the drugs under study was investigated. BGE viscosity is decreased by high temperatures, resulting in a shorter run time. Even while 30 °C produced the best migration times for all drugs, the data lacked high repeatability. As a result, 25 °C was chosen for the analysis.

#### HPLC method

Achieving adequate resolution and satisfactory peak symmetry within a reasonable run time is the most crucial factor in the development of HPLC methods. Many tests were run to optimize the stationary and mobile phases in order to accomplish this goal.

Analytical columns tried in this study were Agilent Zorbax SB-C8 Stable Bond column (4.6 × 250 mm, 5 µm), Agilent ZORBAX Extend-C18 (4.6 × 250 mm, 5 µm) and Waters Symmetry C18 (3.9 × 150 mm, 5 µm). The Agilent ZORBAX Extend-C18 (4.6 × 250 mm, 5 µm) gave the best resolution between the tested drugs in a relatively short run time. Accordingly, it was chosen as the working column for this study. The column was coupled with Agilent ZORBAX Extend-C18 guard column (4.6 × 12.5 mm, 5 µm).

Several mobile phases were evaluated using various proportions of different aqueous phases and organic modifiers adjusted at various pH values. The intended separation was not achieved by the isocratic mobile phase. It was discovered that the optimal mobile phase combination for the assay of this complex mixture was methanol with phosphate buffer at pH 3. Ethanol was tried with phosphate buffer in different eluting modes. Unfortunately, ethanol was not able to separate as many drugs as desired and showed undesired tailed peaks of the eluted drugs. Also, acetonitrile and methanol were tried with phosphate buffer at pH 3 in different gradient elution modes, but with acetonitrile, most drug peaks overlapped and were not well separated. Additionally, various gradient elution programs were tested with 50 mM phosphate buffer pH 3 and methanol. The best chromatograms and shortest run times were achieved with gradient elution starting with a ratio of 70:30 by volume. At 5, 7, and 8 min, methanol was increased to 60, 80, and 90%, respectively. Prior to the next injection, the methanol ratio was brought back to 30%.

Upon investigating the pKa of the studied drugs (Table 1), the effect of mobile phase pH was investigated within the range 3–6 at 1.0 pH unit interval and the best chromatogram was obtained at pH 3. The peaks of AML, VAL, and ATR were overlapped at pH 5, however, the VIC showed a forked peak at pH 6. However, because buffer pH 4 required a longer run time to achieve the same level of peak separation, pH 3 was used for this investigation.

After studying the impact of flow rate, it was discovered that 1 mL/min produced the best results in terms of runtime, peak asymmetry, and column pressure. Lastly, during the chromatographic run, the column temperature was maintained at 25 ºC.

The separation of the studied drugs was performed at 220 nm (Fig. 1d). The quantitative determination for LSP, VAL, ATR and AML was done at 210 nm, whereas for HCT, VIC, BSL and CVL was done at 220 nm.

Within an appropriate run time, the chromatographic conditions previously stated showed almost symmetric peaks and high resolution between the eight drugs. According to the FDA's requirements, system suitability criteria were determined for the analyzed drugs and found to be acceptable (Table S4): N > 2000, α > 1, Rs > 2, k' > 2, and T ≤ 2 [3]. Also, DAD confirms peak purity as the purity angles were below the threshold values as illustrated in Fig. S5.

### Validation of the proposed methods

Validation of the proposed methods was assessed as per the International Conference on Harmonization (ICH) guidelines [78].

#### Linearity and concentration ranges

In MEKC method, the linearity was performed in the concentration ranges of 5–50 µg/mL for HCT, ATR, BSL and AML, 10–100 µg/mL for CPL and VIC, 20–200 µg/mL for VAL. The linearity parameters are collected and summarized in Table 2.

**Table 2:** Characteristic parameters for the regression equations of the proposed MEKC and HPLC methods for the determination of HCT, CPL, LSP, VAL, ATR, VIC, BSL, AML and CVL

Parameters	Proposed Method	HCT	CPL	LSP	VAL	ATR	VIC	BSL	AML	CVL
Detection Wavelength (nm)	MEKC	220	210	210	210	210	220	220	210	220
	HPLC									
Linearity range (µg/mL)	MEKC	5–50	10–100	–	20–200	5–50	10–100	5–50	5–50	–
	HPLC	0.5–50	–	1–50	2–200	0.5–50	0.5–50	1–50	1–50	0.5–50
LOQ (µg/mL)	MEKC	3.93	5.69	–	14.36	3.88	5.86	4.47	4.50	–
	HPLC	0.49	–	0.89	1.72	0.37	0.34	0.69	0.85	0.47
LOD (µg/mL)	MEKC	1.30	1.88	–	4.74	1.28	1.94	1.47	1.49	–
	HPLC	0.16	–	0.29	0.57	0.12	0.11	0.23	0.28	0.16
Intercept	MEKC	0.28	-0.49	–	0.65	− 0.41	0.60	0.21	0.50	–
	HPLC	3.00	–	− 4.67	1.51	2.11	1.94	0.44	0.63	− 2.36
Slope	MEKC	1.80	0.39	–	1.27	1.41	1.72	0.66	0.80	–
	HPLC	81.93	–	17.50	76.40	44.19	47.21	16.34	49.00	44.73
Correlation coefficient	MEKC	0.9998	0.9999	–	0.9998	0.9998	0.9998	0.9996	0.9998	–
	HPLC	0.9999	–	0.9997	0.9999	0.9999	0.9998	0.9998	0.9999	0.9999
^a^S_a_	MEKC	0.71	0.21	–	1.82	0.55	1.01	0.29	0.36	–
	HPLC	4.07	–	1.56	13.14	1.63	1.60	1.13	4.17	2.11
^b^S_b_	MEKC	0.02	3.65 × 10^–3^	–	0.02	0.02	0.02	0.01	0.01	–
	HPLC	0.18	–	0.06	0.15	0.07	0.07	0.05	0.17	0.09
^c^S_y/x_	MEKC	0.87	0.25	–	2.02	0.67	1.20	0.36	0.44	–
	HPLC	7.64	–	2.49	26.85	3.06	2.30	1.80	6.66	3.97
^d^ F	MEKC	5262	11,169	–	5522	5389	9611	4069	4011	–
	HPLC	204,657	–	76,148	256,161	371,451	441,818	127,283	83,643	225,867
Significance F	MEKC	1.90 × 10^–4^	8.95 × 10^–5^	–	1.81 × 10^–4^	1.86 × 10^–4^	1.04 × 10^–4^	2.57 × 10^–4^	2.49 × 10^–4^	–
	HPLC	1.43 × 10^–10^	–	1.05 × 10^–7^	5.71 × 10^–13^	4.35 × 10^–11^	3.07 × 10^–11^	4.86 × 10^–8^	9.12 × 10^–8^	1.18 × 10^−^^10^

^a^Standard deviation of the intercept

^b^Standard deviation of the slope

^c^Standard deviation of the residuals

^d^Variance ratio, equals the mean of squares due to regression divided by the mean of squares about regression (due to residuals)

In HPLC method, the linearity was verified in the range of 0.5–50 µg/mL for four drugs HCT, VIC, CVL and ATR, 1–50 µg/mL LSP, BSL and AML, 2–200 µg/mL for VAL.

Table 2, demonstrates the obtained linearity parameters. Regression calculations for both methods showed good linearity as proved by the correlation coefficient values that are not less than 0.9996. Moreover, the methods showed high F-values providing a steeper regression line and low significance F resulting in minimal scattering of experimental points around the regression line [79].

Many attempts were made to optimize both methods in order to separate the same drugs. Regretfully, CVL was co-eluted with AML and LSP was co-eluted with HCT in the MEKC method. As a result, BSL proved enough as a β-blocker, and CVL was removed. Consequently, AML was retained as a typical case for calcium channel blockers. Furthermore, as both CPL and LSP are non-prodrugs and members of the same class (ACEIs), they were substituted for one another keeping HCT as the most commonly used diuretic found in most combined pharmaceutical formulations with other cardiovascular drugs. Nevertheless, CPL and VIC peaks were overlapped during HPLC optimization, despite numerous attempts to separate the drugs. Ultimately, in the HPLC method it was better to switch out CPL with LSP, a similar drug that was successfully maintained isolated from VIC and the other drugs undergoing research.

#### Detection and quantitation limits

In MEKC method, the LOD and LOQ were in the range of 1.28–4.74 and 3.88–14.36 µg/mL, respectively as illustrated in Table 2. Meanwhile, in HPLC method, the LOD and LOQ were in the range of 0.11–0.57 and 0.34–1.72 µg/mL, respectively as illustrated in Table 2.

#### Accuracy and precision

Three replicates of three concentration levels for each drug were examined at the same time to determine the intra-day precision and accuracy of the suggested procedures. Analyzing the same three concentrations for each drug using three replicate measurements made on three different days allowed for the testing of the inter-day precision. For both approaches, a high degree of precision and accuracy was met, with RSD% and Er% values falling below 2% (Tables S1 & S2).

#### Specificity

In both methods, the analysis of different samples containing different concentrations of each drug using the proposed methods showed good percentage recoveries indicating good specificity of the method (Tables S1 & S2). Furthermore, the application of the proposed methods for the determination of the tested drugs in their single and combined dosage forms, without the interference of the excipients, demonstrated the specificity of the method (Table 3 and S3).

**Table 3:** Assay results for the determination of HCT, CPL, LSP, VAL, ATR, VIC, BSL, AML and CVL in their combined dosage forms using the proposed MEKC and HPLC methods (n = 5)

Dosage forms	Proposed MEKC method		Proposed HPLC method		Reference method^c^	
Co-tareg® tablets^a^	VAL	HCT	VAL	HCT	VAL	HCT
Mean % recovery ± SD	98.91 ± 1.69	101.54 ± 0.68	99.47 ± 0.74	99.56 ± 0.91	99.5 ± 1.1	100.8 ± 0.45
RSD%	1.71	0.67	0.75	0.91	1.11	0.45
Er (%)	− 1.09	1.54	− 0.53	− 0.44	− 0.50	0.80
t^b^	0.50	0.91	0.04	2.11		
F^b^	2.37	2.29	2.19	4.06		

Exforge® tablets^a^	AML	VAL	AML	VAL	AML	VAL
Mean % recovery ± SD	99.33 ± 0.45	99.40 ± 0.48	101.04 ± 1.19	99.13 ± 0.51	99.90 ± 1.10	100.56 ± 1.10
RSD%	0.45	0.49	1.18	0.52	1.10	1.09
Er (%)	− 0.67	− 0.60	1.04	− 0.87	− 0.1	0.56
t^b^	0.83	1.68	1.22	2.04		
F^b^	6.02	5.15	1.18	4.60		

Capozide® tablets^a^	CPL	HCT	CPL	HCT	CPL	HCT
Mean % recovery ± SD	99.99 ± 1.31	100.16 ± 0.68			98.30 ± 1.20	100.10 ± 0.92
RSD%	1.31	0.68			1.22	0.92
Er (%)	− 0.01	0.16			− 1.70	0.10
t^b^	1.65	0.08				
F^b^	1.19	1.83				

Zestoretic® tablets^a^	LSP	HCT	LSP	HCT	LSP	HCT
Mean % recovery ± SD			99.65 ± 1.02	100.61 ± 0.50	98.40 ± 0.43	100.00 ± 0.24
RSD%			1.02	0.50	0.44	0.24
Er (%)			− 0.35	0.61	− 1.60	0
t^b^			1.96	1.89		
F^b^			5.58	4.36		

Concor plus® tablets^a^	BSL	HCT	BSL	HCT	BSL	HCT
Mean % recovery ± SD	100.22 ± 1.52	99.78 ± 0.72	98.17 ± 1.87	100.04 ± 0.59	100.70 ± 0.90	99.60 ± 1.00
RSD%	1.52	0.72	1.90	0.59	0.90	1.00
Er (%)	0.22	-0.22	− 1.83	0.04	0.70	− 0.4
t^b^	0.47	0.26	2.11	0.66		
F^b^	2.86	1.95	4.30	2.91		

Caduet® tablets ^a^	AML	ATR	AML	ATR	AML	ATR
Mean % recovery ± SD	100.28 ± 1.90	100.16 ± 0.94	99.15 ± 1.22	101.02 ± 0.59	99.05 ± 0.99	100.19 ± 0.51
RSD%	1.90	0.94	1.23	0.58	0.99	0.50
Er (%)	0.28	0.16	− 0.85	1.02	− 0.95	0.19
t^b^	0.10	0.05	0.68	1.84		
F^b^	3.68	3.46	1.52	1.36		

Co-dilatrol® tablets ^a^	CVL	HCT	CVL	HCT	CVL	HCT
Mean % recovery ± SD			99.53 ± 1.22	100.80 ± 1.17	99.91 ± 0.51	99.90 ± 0.72
RSD%			1.23	1.16	0.51	0.72
Er (%)			− 0.47	0.80	− 0.09	0.1
t^b^			0.49	1.14		
F^b^			5.73	2.62		

Exforge HCT® tablets^a^	AML	VAL	HCT	AML	VAL	HCT	AML	VAL	HCT
Mean % recovery ± SD	99.45 ± 1.24	99.43 ± 0.53	99.75 ± 1.36	100.57 ± 1.92	100.08 ± 0.55	100.70 ± 1.16	100.55 ± 0.85	100.65 ± 1.23	99.36 ± 1.07
RSD%	1.24	0.53	1.36	1.91	0.55	1.16	0.85	1.22	1.08
Er (%)	− 0.55	− 0.57	− 0.25	0.57	0.08	0.70	0.55	0.65	− 0.64
t^b^	1.26	1.58	0.39	0.01	0.73	1.47			
F^b^	2.14	5.46	1.62	5.10	4.94	1.19			

^a^Co-tareg® tablets (80 mg VAL and 12.5 mg HCT/tablet), Exforge® tablets (10 mg AML and 160 mg VAL/tablet), Capozide® tablets (50 mg CPL and 25 mg HCT/tablet), Zestoretic® tablets (20 mg LSP and 12.5 mg HCT/tablet), Concor plus® tablets (10 mg BSL and 25 mg HCT/tablet), Caduet® tablets (5 mg AML and 10 mg ATR/tablet), Co-dilatrol® tablets (25 mg CVL and 12.5 mg HCT/tablet) and Exforge HCT ® tablets (10 mg AML,160 mg VAL and 12.5 mg HCT/tablet)

^b^ Theoretical values of t and F are 2.31 and 6.39, respectively, at 95% confidence limit (n = 5)

^c^ Reference methods are: Co-tareg® [9], Exforge® [84], Exforge HCT® [31], Capozide® [9], Zestoretic® [86], Concor plus® [87], Caduet® [88] and Co-dilatrol® [85]

**Fig. 2:**
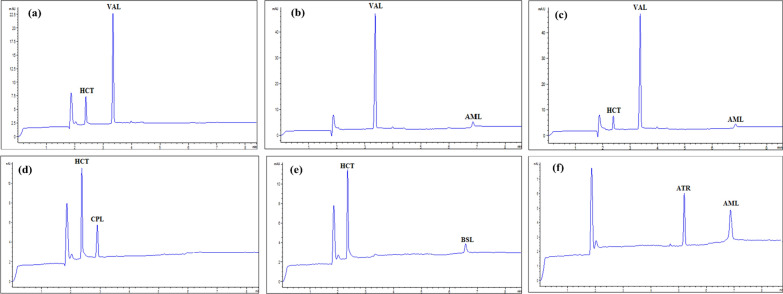
Electropherograms of HCT, CPL, VAL, ATR, VIC, BSL and AML prepared from their combined dosage forms; **a** Co-tareg®, **b** Exforge®, **c** Exforge HCT®, **d** Capozide®, **e** Concor plus® and **f** Caduet® respectively, measured at 220 nm

**Fig. 3:**
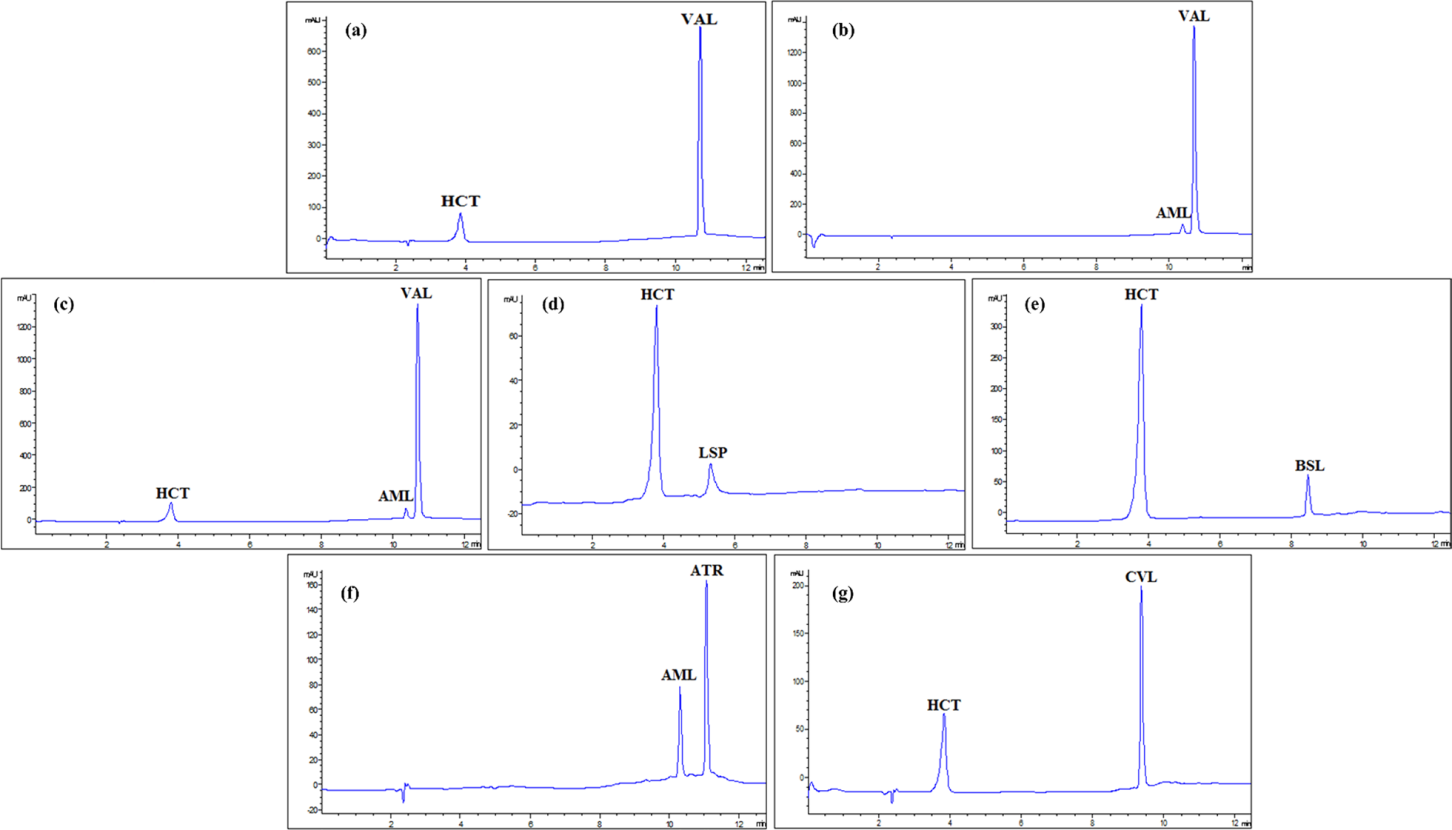
Chromatograms of HCT, LSP, VIC, BSL, CVL, AML, VAL and ATR prepared from their combined dosage forms; **a** Co-tareg®, **b** Exforge®, **c** Exforge HCT®, **d** Zestoretic®, **e** Concor plus®, **f** Caduet® and Co-dilatrol®, respectively, measured at 220 nm

The method specificity was demonstrated by similar electropherograms (MEKC method) and chromatograms (HPLC method) and essentially unchanged migration/retention durations of the drugs under study obtained from the standard solutions when compared to that of dosage form solutions (Figs. 1a, d, 2, 3, S2 & S3).

In addition, the peak purity of all drugs was checked by using a G1315 C/D and G1365 C/D photo diode array detector (DAD) (Fig. S4 & S5). The purity angle in every sample was found to be under the purity threshold limit, indicating that no extra peaks were co-eluting with any of the analytes and demonstrating the proposed methods' capability to evaluate the target analyte even in the presence of possible interferences.

#### Robustness

Deliberate adjustments were made to the methods' parameters, the robustness was assessed by computing the SD and RSD of both peak area ratios and migration or retention times. Triplicate injections were used for analysis, and each time, the parameters under study underwent a single modification. The proposed methods were robust since the examined modifications had no significant impact on the studied drugs' peak area ratios or migration periods, as indicated by RSD% values that were less than or equal to ± 2, as indicated in Table S5.

#### Stability of solutions

Throughout the analysis time, the stability of the nine drugs under study in their working solutions was examined. For two, four, and six hours, the working standard solutions were made and stored at room temperature. The solutions were examined using the proposed methods at various intervals of time. The drugs were stable under these conditions, according to analysis, and no alterations were found. The peak area values and migration or retention times of the analyzed drugs showed no significant changes with %RSD values below 2%. When stored at 4 °C, stock solutions were likewise found to remain stable for at least two weeks. By applying the proposed methods to analyze the calculated concentrations of freshly made solutions and those stored for two weeks, the difference was determined to be less than 2%.

### Assay of tablets dosage forms

The proposed methods were applied for the assay of the nine studied drugs in their single, binary and ternary dosage forms available in the Egyptian market. Sample preparation was done as described in Sect. “Analysis of pharmaceutical preparations” and then aliquots were diluted before injection to give a final concentration within the specified linearity range. Every drug was eluted at the appropriate migration or retention times. Neither the dosage form matrix nor any of the inactive components showed any interference peaks (Figs. 2, 3, S2 & S3). Recoveries and %RSD were calculated and illustrated in Tables S3 & 3, showing accepted values.

The drugs in their single dosage forms and in their combined dosage forms were compared to reported reference methods. The assay results revealed satisfactory accuracy and precision as indicated from % recovery, SD, and % RSD values (Table S3 & 3). Recovery data obtained from the proposed methods was statistically compared to the reported methods using the Student’s t- and the variance ratio F-tests to assess accuracy and precision, respectively. In both tests, the calculated values did not exceed the theoretical ones at the 95% confidence level (Table S3 & 3).

### Bioanalytical validation of the proposed methods

In order for the proposed methods to be applied for the analysis of various biological fluids, the proposed methods were validated regarding linearity, LLOQ, accuracy, precision, recovery, stability and selectivity as per the FDA Bioanalytical Method Validation [80].

#### Calibration curve

To create calibration curves over the concentration ranges shown in Table 4, various aliquots of HCT, CPL, LSP, VAL, ATR, VIC, BSL, AML, and CVL were spiked into 100 µL of rat plasma. The calibration curves were constructed using the internal standard (IS) method by plotting the ratios of the studied drugs peak areas to IS peak areas against the corresponding concentrations. The concentrations, 100 and 20 µg/mL of ATR were chosen to be IS for all other drugs while 100 and 20 µg/mL HCT were the IS for ATR in MEKC and HPLC, respectively (Table 4).

**Table 4:** Characteristic parameters for the regression equations of the proposed MEKC and HPLC methods for the determination of HCT, CPL, LSP, VAL, ATR, VIC, BSL, AML and CVL in rat plasma

Parameters		HCT	CPL	LSP	VAL	ATR	VIC	BSL	AML	CVL
Detection Wavelength (nm)	MEKC	220	210	210	210	210	220	220	210	220
	HPLC									
Linearity range (µg/mL)	MEKC	50–1000	100–1000		50–2000	50–1000	10–1000	50–1000	50–1000	–
	HPLC	1–100	−	1–100	2–200	5–100	2–100	5–100	1–100	5–100
LLOQ (µg/mL)	MEKC	50	100	−	50	50	50	50	50	–
	HPLC	1	−	1	2	5	2	5	1	5
LOD (µg/mL)	MEKC	25	50	−	20	30	5	30	25	–
	HPLC	0.40	−	0.57	1.46	2.75	1.29	1.23	0.45	2.63
Intercept	MEKC	− 2.03 × 10^–1^	− 1.05 × 10^–1^	−	1.50 × 10^–1^	4.35 × 10^–2^	5.76 × 10^–2^	− 4.76 × 10^–2^	− 5.77 × 10^–2^	–
	HPLC	1.83 × 10^–2^	−	8.31 × 10^–3^	2.81 × 10^–2^	− 1.35 × 10^–2^	4.50 × 10^–2^	7.06 × 10^–4^	8.36 × 10^–3^	2.70 × 10^–1^
Slope	MEKC	1.55 × 10^–2^	7.35 × 10^–3^	−	1.10 × 10^–2^	7.35 × 10^–3^	2.05 × 10^–2^	9.79 × 10^–3^	8.32 × 10^–3^	–
	HPLC	1.89 × 10^–1^	−	4.63 × 10^–2^	4.79 × 10^–2^	1.04 × 10^–1^	1.10 × 10^–1^	3.97 × 10^–2^	1.12 × 10^–1^	2.56 × 10^–1^
Correlation coefficient	MEKC	0.9996	0.9997	–	0.9999	0.9998	0.9998	0.9999	0.9996	–
	HPLC	0.9999	–	0.9999	0.9999	0.9995	0.9998	0.9999	0.9999	0.9996
^a^S_a_	MEKC	9.40 × 10^–2^	4.49 × 10^–2^	–	4.53 × 10^–2^	2.31 × 10^–2^	6.82 × 10^–2^	2.93 × 10^–2^	4.85 × 10^–2^	–
	HPLC	2.51 × 10^–2^	–	8.78 × 10^–3^	2.33 × 10^–2^	9.56 × 10^–2^	4.74 × 10^–2^	1.63 × 10^–2^	1.66 × 10^–2^	2.24 × 10^–1^
^b^S_b_	MEKC	2.06 × 10^–4^	9.13 × 10^–5^	–	5.49 × 10^–5^	5.07 × 10^–5^	1.60 × 10^–4^	6.43 × 10^–5^	1.06 × 10^–4^	–
	HPLC	5.82 × 10^–4^	–	2.03 × 10^–4^	2.68 × 10^–4^	1.87 × 10^–3^	1.02 × 10^–3^	3.20 × 10^–4^	3.85 × 10^–4^	4.39 × 10^–3^
^c^S_y/x_	MEKC	1.64 × 10^–1^	6.66 × 10^–2^	–	9.57 × 10^–2^	4.03 × 10^–2^	1.37 × 10^–1^	5.11 × 10^–2^	8.47 × 10^–2^	–
	HPLC	5.20 × 10^–2^	–	1.82 × 10^–2^	4.76 × 10^–2^	1.47 × 10^–1^	8.62 × 10^–2^	2.51 × 10^–2^	3.44 × 10^–2^	3.45 × 10^–1^
^d^F	MEKC	5664	6479	–	39,766	20,996	16,495	23,161	6113	–
	HPLC	105,525	–	51,894	32,109	3099	11,752	15,401	84,110	3395
Significance F	MEKC	7.85 × 10^–9^	1.43 × 10^–7^	–	1.07 × 10^–12^	2.97 × 10^–10^	1.50 × 10^–11^	2.32 × 10^–10^	6.49 × 10^–9^	–
	HPLC	5.25 × 10^–12^		3.09 × 10^–11^	1.03 × 10^–10^	1.28 × 10^–5^	4.34 × 10^–8^	1.15 × 10^–6^	9.25 × 10^–12^	1.11 × 10^–5^

^a^Standard deviation of the intercept

^b^Standard deviation of the slope

^c^Standard deviation of the residuals

^d^Variance ratio, equals the mean of squares due to regression divided by the mean of squares about regression (due to residuals)

#### Limit of detection (LOD) and lower limit of quantitation (LLOQ)

Five replicates were used to analyze the LOD and LLOQ, and the analyte's finding was compared to the blank response. (Table 4). LOD and LLOQ were calculated using signal-to-noise (S/N) ratio of 3:1 and 5:1, respectively, to that of the blank response. LODs as low as 5 µg/mL and 0.4 µg/mL were obtained upon using MEKC and HPLC, respectively, indicating the sensitivity of the proposed methods.

#### Accuracy and precision

The accuracy and precision of the proposed methods were validated. For intra-day and inter-day assay validation, six replicates of each of the four quality control points, LLOQ, LOQ, MQC, and HQC, within the calibration range of each drug were determined on the same day and three separate days later.

A newly developed calibration curve was used every day to determine the concentration of the drugs under study. The precision was measured in terms of percentage coefficient of variation (%RSD), while the accuracy was measured in terms of mean percentage recoveries and percentage error of the mean (%Er) (Tables S6 & S7).

#### Stability

A recovery of 85–115% of the original concentrations of the studied drugs in plasma confirms their stability. Achieving satisfactory recovery and RSD values for all the stability trials (Tables S8 & S9) of the drugs under study in rat plasma indicates their stability under various settings.

### Application of different co-administered dosage forms of the studied drugs with vincamine in spiked plasma samples

**Fig. 4:**
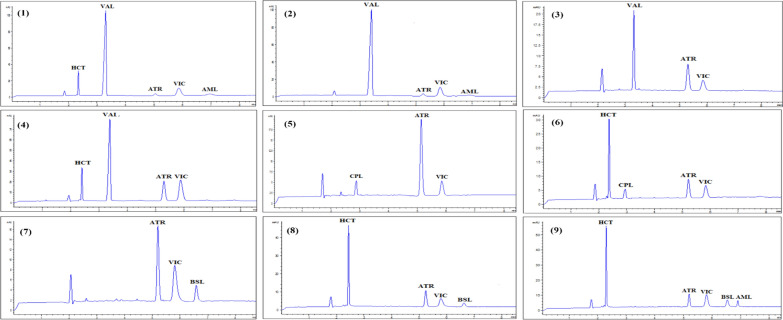
Electropherograms of HCT, CPL, VAL, ATR, VIC, BSL and AML in their possible co-administered combined dosage forms in rat plasma; **1** Exforge HCT®/Ator 10®/Brain-OX®, **2** Exforge®/Ator 10®/Brain-OX®, **3** Tareg 80®/Ator 10®/Brain-OX®, **4** Co-tareg ®/Ator 10®/Brain-OX®, **5** Capoten 50®/Ator 10®/Brain-OX®, **6** Capozide®/Ator 10®/Brain-OX®, **7** Concor 10®/Ator 10®/Brain-OX®, **8** Concor plus®/Ator 10®/Brain-OX® and **9** Caduet®/Concor plus®/Brain-OX®, measured at 220 nm

**Fig. 5:**
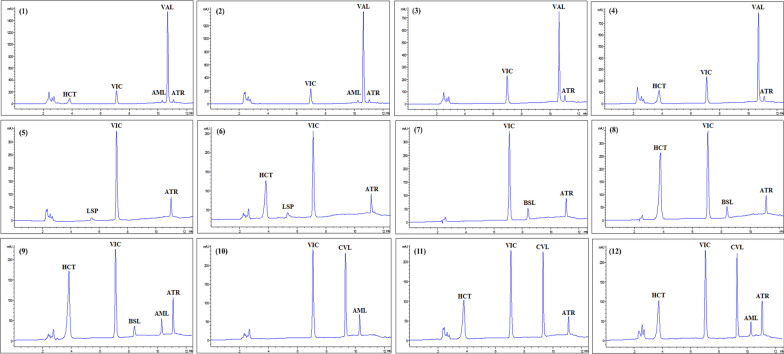
Chromatograms of HCT, LSP, VIC, BSL, CVL, AML, VAL and ATR in their possible co-administered combined dosage forms in rat plasma; **1** Exforge HCT®/Ator 10®/Brain-OX®, **2** Exforge®/Ator 10®/Brain-OX®, **3** Tareg 80®/Ator 10®/Brain-OX®, **4** Co-tareg ®/Ator 10®/Brain-OX®, **5** Zestril 10®/Ator 10®/Brain-OX®, **6** Zestoretic®/Ator 10®/Brain-OX®, **7** Concor 10®/Ator 10®/Brain-OX®, **8** Concor plus®/Ator 10®/Brain-OX®, **9** Caduet®/Concor plus®/Brain-OX®, **10** Norvasc 10®/Dilatrol 25®/Brain-OX®, **11** Co-dilatrol ®/Ator 10®/Brain-OX® and **12** Co-dilatrol ®/Caduet®/Brain-OX® measured at 220 nm

The suggested methods were effectively used to determine the administered cardiovascular drugs in rat plasma in combination with vincamine. In this work, the analysis of different possible combinations of co-administered dosage forms indicated for treating various CVDs together with VIC was performed and demonstrated good recovery rates with relative standard deviations within acceptable limits, ensuring precise and accurate quantification (Table 5, Fig. 4 and 5). The analyzed samples were prepared using the drugs’ concentrations within the ratio of the co-administered dosage forms. The prepared ratios of the co-administered drugs in their dosage forms were, combination 1; 1, 16, 1.25, 1, 3 for AML, VAL, HCT, ATR, VIC, combination 2; 1, 16, 1, 3 for AML, VAL, ATR, VIC, combination 3; 8, 1, 3 for VAL, ATR, VIC, combination 4; 8, 1.25, 1, 3 for VAL, HCT, ATR,VIC, combination 5; 5, 1, 3 for CPL, ATR, VIC, combination 6; 5, 2.5, 1, 3 for CPL, HCT, ATR, VIC, combination 7; 1, 1, 3 for LSP, ATR, VIC, combination 8; 2, 1.25, 1, 3 for LSP, HCT, ATR, VIC, combination 9; 1, 1, 3 for BSL, ATR, VIC, combination 10; 1, 2.5, 1, 3 for BSL, HCT, ATR, VIC, combination 11; 1, 2.5, 0.5, 1, 3 for BSL, HCT, AML, ATR, VIC, combination 12; 1, 2.5, 3 for AML, CVL, VIC, combination 13; 2.5, 1.25, 1, 3 for CVL, HCT, ATR, VIC, combination 14; 2.5, 1.25, 0.5, 1, 3 for CVL, HCT, AML, ATR, VIC, respectively (Table 5, Fig. 4 and 5).

**Table 5:** Application of MEKC and HPLC methods in the determination of HCT, CPL, LSP, VAL, ATR, VIC, BSL, AML and CVL in their possible co-administered dosage forms in rat plasma (n = 5)

Method		MEKC			HPLC		
Dosage forms^a^		Mean % recovery ± SD	RSD%	Er (%)	Mean % recovery ± SD	RSD%	Er (%)
Combinations							
Combination 1							
Exforge HCT®	AML	100.23 ± 6.05	6.03	0.23	97.21 ± 3.48	3.58	− 2.79
	VAL	98.40 ± 2.61	2.66	− 1.60	101.31 ± 1.23	1.21	1.31
	HCT	100.25 ± 2.36	2.36	0.25	96.28 ± 4.04	4.20	− 3.72
Ator 10®	ATR	103.47 ± 2.83	2.74	3.47	104.01 ± 5.50	5.29	4.01
Brain OX®	VIC	99.67** ± **4.29	4.31	− 0.33	100.64 ± 4.00	3.98	0.64
Combination 2							
Exforge®	AML	101.03 ± 5.68	5.62	1.03	99.09 ± 4.51	4.56	− 0.91
	VAL	97.07 ± 3.44	3.54	− 2.93	102.19 ± 0.50	0.49	2.19
Ator 10®	ATR	104.83** ± **2.08	1.98	4.83	103.49 ± 7.83	7.57	3.49
Brain OX®	VIC	102.92 ± 1.62	1.58	2.92	99.80 ± 1.84	1.84	− 0.20
Combination 3							
Tareg®	VAL	99.07 ± 7.42	7.49	− 0.93	99.83 ± 3.12	3.12	− 0.17
Ator 10®	ATR	102.11 ± 4.78	4.68	2.11	103.94 ± 6.03	5.81	3.94
Brain OX®	VIC	98.59 ± 4.08	4.14	− 1.41	99.02 ± 2.86	2.89	− 0.98
Combination 4							
Co-tareg®	VAL	99.45 ± 3.67	3.69	− 0.55	100.32 ± 1.72	1.71	0.32
	HCT	98.70 ± 1.86	1.88	− 1.30	100.48 ± 2.66	2.65	0.48
Ator 10®	ATR	100.75 ± 5.50	5.46	0.75	101.71 ± 5.45	5.36	1.71
Brain OX®	VIC	99.57 ± 3.25	3.27	− 0.43	101.70 ± 0.80	0.79	1.70
Combination 5							
Capoten®	CPL	100.26 ± 2.57	2.57	0.26			
Ator 10®	ATR	101.68 ± 3.40	3.34	1.68			
Brain OX®	VIC	98.20 ± 4.08	4.16	− 1.8			
Combination 6							
Capozide ®	CPL	99.36 ± 1.63	1.64	− 0.64			
	HCT	102.48 ± 1.84	1.80	2.48			
Ator 10®	ATR	101.68 ± 2.72	2.68	1.68			
Brain OX®	VIC	100.90 ± 4.96	4.91	0.90			
Combination 7							
Zestril 10®	LSP				97.28 ± 5.12	5.27	− 2.72
Ator 10®	ATR				104.31 ± 5.61	5.38	4.31
Brain OX®	VIC				100.17 ± 2.37	2.37	0.17
Combination 8							
Zestoretic®	LSP				98.64 ± 2.13	2.16	− 1.36
	HCT				98.09 ± 0.74	0.75	− 1.91
Ator 10®	ATR				102.60 ± 7.07	6.89	2.60
Brain OX®	VIC				103.38 ± 3.23	3.13	3.38
Combination 9							
Concor 10®	BSL	101.66 ± 2.04	2.01	1.66	98.42 ± 2.57	2.61	− 1.58
Ator 10®	ATR	100.77 ± 6.85	6.80	0.77	99.09 ± 3.91	3.94	− 0.91
Brain OX®	VIC	100.25 ± 4.24	4.22	0.25	98.59 ± 2.45	2.48	− 1.41
Combination 10							
Concor plus ®	BSL	96.89 ± 4.25	4.39	− 3.11	102.90 ± 4.23	4.11	2.90
	HCT	102.96 ± 2.61	2.53	2.96	103.12 ± 2.83	2.74	3.12
Ator 10®	ATR	97.14 ± 3.14	3.24	− 2.86	102.50 ± 4.61	4.50	2.50
Brain OX®	VIC	98.74 ± 3.25	3.29	− 1.26	99.20 ± 5.73	5.77	− 0.80
Combination 11							
Concor plus®	BSL	100.54 ± 3.59	3.57	0.54	101.44 ± 3.85	3.79	1.44
	HCT	98.96 ± 1.97	1.99	− 1.04	103.03 ± 2.13	2.07	3.03
Caduet®	AML	105.15 ± 4.81	4.57	5.15	104.41 ± 2.26	2.16	4.41
	ATR	101.20 ± 1.36	1.34	1.20	100.20 ± 3.72	3.72	0.20
Brain OX®	VIC	98.05 ± 2.98	3.04	− 1.95	99.10 ± 4.80	4.85	− 0.90
Combination 12							
Norvasc 10 ®	AML				99.54 ± 2.57	2.58	− 0.46
Dilatrol 25 ®	CVL				100.61 ± 4.42	4.40	0.61
Brain OX®	VIC				100.21 ± 2.31	2..31	0.21
Combination 13							
Co-dilatrol®	CVL				99.70 ± 2.67	2.67	− 0.30
	HCT				99.96 ± 3.08	3.08	− 0.04
Ator 10®	ATR				99.09 ± 2.88	2.90	− 0.91
Brain OX®	VIC				97.58 ± 1.39	1.42	− 2.42
Combination 14							
Co-dilatrol®	CVL				100.71 ± 1.75	1.73	0.71
	HCT				100.63 ± 2.02	2.00	0.63
Caduet®	AML				99.41 ± 2.93	2.95	− 0.59
	ATR				101.48 ± 5.51	5.43	1.48
Brain OX®	VIC				98.69 ± 4.62	4.68	− 1.31

^a^In each combination, the drugs were determined in their ratio as per the co-administered dosage forms

The analysis was completed in a reasonable time frame, 7 and 11.5 min, for MEKC and HPLC, respectively, showcasing the efficiency of the techniques for simultaneous drug determination in biological matrices (Fig. 1c and f).

### Greenness assessment of the proposed methods

The greenness of analytical methods is increasingly assessed using tools such as EcoScale, the Green Analytical Procedure Index (GAPI), and the Analytical GREEnness (AGREE) metric. EcoScale evaluates the environmental impact of analytical methods based on factors like reagent toxicity and energy consumption, assigning a score to reflect sustainability criteria such as sample preparation, reagents, and instrumentation [81]. Unfortunately, ecoscale does not provide information regarding the risks' structures or the reasons for the analytical procedure's environmental impact, like the usage of solvents or other reagents, occupational hazards, or waste generation. AGREE synthesizes multiple green chemistry principles into a single, user-friendly score [82]. It is characterized by automation and highlighting the weak points that need further optimization in the suggested analytical method. GAPI provides a comprehensive, visual representation of method greenness, considering the assessment and comparison of eco-friendly analytical procedures [83]. It can be used as a semi-quantitative tool for laboratory practice. Both AGREE and GAPI are represented as colored pictograms that are easily interpreted.

In Table S10, the proposed methods were compared to eight different reported methods [9, 31, 76, 84–88] to compare their greenness. The proposed MEKC method showed excellent green analysis by having an analytical Eco-scale of 89 (above 75) and the highest AGREE score (0.89). GAPI pictogram for both proposed methods were comparable to the reference methods, where proposed MEKC surpassed all the methods in the waste amount. This indicates the superior greenness of MEKC method.

### Blueness assessment of the proposed methods

The proposed analytical methods, designed for the separation of various cardiovascular drugs alongside vincamine, emphasizes environmental sustainability by incorporating the Blue Applicability Grade Index (BAGI) [89]. This index evaluates the method's water and energy usage, toxicity, and waste production, ensuring a minimal environmental footprint. By achieving a high Blue Index rating, the method not only proves to be efficient and reliable for drug analysis but also aligns with eco-friendly practices. This approach underscores the importance of green chemistry principles in modern analytical techniques. In Fig. S6, the proposed methods showed dark blue color showing good compliance of the analytical methods to the criteria. Both proposed methods scored 80 revealing excellent performance of the method. In comparison with the reported reference methods, our proposed methods showed comparable score in BAGI to two reported reference HPLC methods [9, 31]. This score outperformed other reference methods [84–88], whereas another HPLC reported method [76] showed the least score of 75 as illustrated in Fig. S6.

### Whiteness assessment of the proposed methods

Analytical (red) and practical (blue) factors are two more important factors that White Analytical Chemistry (WAC) considers when evaluating the method's quality. The coherence and synergy of the analytical, ecological, and practical elements are demonstrated by a white analytical approach in connection to the RGB color model, which implies that the combination of red, green, and blue light beams produces the appearance of whiteness.

The proposed methods along with other reported methods [9, 31, 76, 84–88] were evaluated for their whiteness using the multicriteria RGB 12 model [90]. The resulted data was illustrated in Fig. S7. The proposed MEKC and HPLC methods showed superior performance over the reference methods.

The greenness, blueness, and whiteness approaches demonstrated the superiority of both the MEKC and HPLC procedures. Both techniques separated the drugs in a comparatively short amount of time during analysis by using less hazardous reagents in modest amounts. The suggested methods received good score in these evaluations because of their capacity to separate multicomponent samples with various chemical classes. Additionally, the suggested approaches outperformed previously published reference methods in every area of WAC due to their reduced power consumption and straightforward sample preparation.

To summarize the evaluation of greenness, blueness, and whiteness, our suggested MEKC method is a superior white and green method compared to other documented chromatographic and spectrophotometric methods. In addition, this suggested method has unique advantages in terms of high separation power with quick analysis times (7 min), small amounts of toxic reagents with minimum energy and waste consumption that have little to no detrimental effects on the environment or public health. On the other hand, the multianalyte analysis of eight medications using our suggested HPLC was completed in less than 12 min and received good marks for greenness, blueness, and whiteness.

The proposed MEKC method showed high capability of simultaneous analysis of seven drugs in relatively short time (7 min) with high resolution (Fig. 1). It is characterized by high green, blue and white profile with high throughput analysis per hour using minimal amount of solvents and waste as well (Table S10, Fig. S6 & S7). On the other side, proposed HPLC method showed higher sensitivity as per lower detection and quantitation limits (Tables 2 and 4). Moreover, eight drugs were simultaneously determined using the HPLC method in less than 12 min (Fig. 1) with high selectivity and reproducibility. Additionally, the HPLC method showed high score in WAC approaches (Table S10, Fig. S6 &S7).

Our proposed methods, MEKC and HPLC, showed their efficacy for simultaneous analysis of various cardiovascular drugs with the nutraceutical, vincamine, in single and combined pharmaceutical formulations in reasonable time frame with good WAC profile. In addition to their applicability in biological matrices; plasma. This highlights the methods’ suitability for possible routine use in pharmaceutical analysis.

## Conclusion

In conclusion, the proposed method includes the determination of a range of cardiovascular drugs, each representing a different drug class, alongside the nutraceutical vincamine. This comprehensive approach allows the simultaneous analysis of various drug classes in a single method.

The proposed analytical methods have demonstrated significant potential for routine use in the analysis of pharmaceutical formulations either single or combined or co-administered multiple drug therapy. These methods offer a cost-effective, time-efficient solution that ensures reliable and accurate separation and quantification of multiple drugs within a short analysis time. Their eco-friendly approach aligns with current green chemistry principles, making these methods a valuable tool for modern pharmaceutical analysis and enhancing its efficiency and sustainability.

Nutraceuticals are increasingly important due to their potential health benefits and are often co-administered with other pharmaceuticals. This necessitates the development of analytical methods to study these combinations. Identifying methods that can simultaneously analyze nutraceuticals and drugs is crucial for understanding their synergistic or antagonistic effects. Future research should focus on these interactions to optimize therapeutic outcomes and ensure safety in combined usage. The proposed methods fill a significant gap as no previous techniques have successfully separated vincamine in the presence of other cardiovascular drugs.

This study paves the way for future research into their potential interactions and combined use in clinical settings, ensuring effective and safe therapeutic regimens.

## Supplementary Information

Supplementary Material 1.

## Data Availability

All data generated or analysed during this study are included in this published article [and its supplementary information files].
