# Supplementary material for: Simple simultaneous analysis of various cardiovascular drug mixtures with vincamine: comparative eco-friendly assessment
Source: BMC Chem. 2024 Oct 10;18(1):197. doi: 10.1186/s13065-024-01303-2 (PMC11468470; doi:10.1186/s13065-024-01303-2)
Supplement: Supplementary file 1 — Supplementary Material 1. [file 13065_2024_1303_MOESM1_ESM.pdf]

Table S1: Intra-day and Inter-day Precision and Accuracy for the determination of HCT, CPL, VAL, ATR, VIC, BSL and AML using the proposed MEKC method

Precision and Accuracy	Intra-day				Inter-day			
	Nominal Concentration (µg/mL)	Mean % Recovery ± SD	RSD%	Er%	Nominal Concentration (µg/mL)	Mean % Recovery ± SD	RSD%	Er%
HCT	10	99.81±1.67	1.67	-0.19	10	100.74 ±1.79	1.78	0.74
	20	101.06±1.43	1.41	1.06	20	101.24±1.16	1.15	1.24
	50	100.13±1.23	1.23	0.13	50	99.69±0.76	0.76	-0.31
CPL	15	100.21±1.73	1.73	0.21	15	100.79±1.00	1.00	0.79
	30	99.43±0.87	0.87	-0.57	30	100.30±1.73	1.73	0.30
	75	98.62±1.73	1.75	-1.38	75	98.62±1.90	1.93	-1.38
VAL	50	99.83±0.55	0.55	-0.17	50	100.09±0.69	0.69	0.09
	100	100.58±0.36	0.35	0.58	100	99.84±0.36	0.36	-0.16
	150	100.16±0.48	0.48	0.16	150	99.99±0.37	0.37	-0.01
ATR	10	101.16±1.08	1.08	1.16	10	100.68±1.79	1.78	0.68
	20	99.69±1.48	1.47	-0.31	20	100.04±0.89	0.89	0.04
	50	100.42±1.26	1.26	0.42	50	99.33±0.71	0.72	-0.67
VIC	15	99.94±1.75	1.75	-0.06	15	99.55±1.19	1.19	-0.45
	30	100.39±1.21	1.21	0.39	30	100.19±0.89	0.89	0.19
	75	100.86±0.59	0.58	0.86	75	101.12±0.16	0.15	1.12
BSL	10	100.22±1.52	1.52	0.22	10	99.71±1.76	1.76	-0.29
	20	99.06±1.91	1.93	-0.94	20	100.58±1.91	1.90	0.58
	50	100.29±1.44	1.43	0.29	50	100.49±1.37	1.37	0.49
AML	10	98.63±1.90	1.93	-1.37	10	99.04±1.44	1.45	-0.96
	20	101.31±1.64	1.62	1.31	20	99.86±1.90	1.90	-0.14
	50	99.36±1.12	1.13	-0.64	50	100.85±0.52	0.51	0.85

[a] **Mean ± SD for three determinations.**

[b] **Percentage relative standard deviation**

[c] **Percentage relative error.**

Table S2: Intra-day and Inter-day Precision and Accuracy for the determination of HCT, LSP, VIC, BSL, CVL, AML, VAL and ATR and using the proposed HPLC method

Precision and Accuracy	Intra-day				Inter-day			
	Nominal Concentration (µg/mL)	Mean % Recovery ± SD	RSD%	Er%	Nominal Concentration (µg/mL)	Mean % Recovery ± SD	RSD%	Er%
**HCT**	1	100.43±1.99	1.98	0.43	1	100.38 ±0.50	0.50	0.38
	20	100.58±1.00	1.00	0.58	20	99.61±0.34	0.34	-0.39
	50	98.69±1.67	1.69	-1.31	50	100.55±1.00	1.00	0.55
**LSP**	5	100.27±0.44	0.44	0.27	5	100.23±1.69	1.68	0.23
	20	99.83±0.39	0.39	-0.17	20	100.94±1.40	1.40	0.94
	50	99.99±0.46	0.46	-0.01	50	100.12±0.24	0.24	0.12
**VIC**	1	101.11±1.12	1.11	1.11	1	99.83±0.66	0.66	-0.17
	20	99.61±0.34	0.34	-0.39	20	100.04±0.48	0.48	0.04
	50	100.24±0.85	0.85	0.24	50	100.27±0.62	0.62	0.27
**BSL**	5	98.63±1.90	1.92	-1.37	5	98.61±0.69	0.70	-1.39
	20	101.31±1.64	1.62	1.31	20	99.02±0.71	0.72	-0.98
	50	99.36±1.12	1.13	-0.64	50	100.84±0.53	0.52	0.84
**CVL**	1	99.37±0.87	0.87	-0.63	1	101.15±0.87	0.86	1.15
	20	100.24±0.77	0.77	0.24	20	99.45±0.87	0.87	-0.55
	50	100.58±0.94	0.93	0.58	50	98.62±1.95	1.98	-1.38
**AML**	5	101.08±1.18	1.17	1.08	5	99.64±1.13	1.13	-0.36
	20	99.63±1.45	1.46	-0.37	20	100.19±1.11	1.11	0.19
	50	100.92±0.92	0.91	0.92	50	98.70±0.67	0.68	-1.30
**VAL**	10	100.70±0.90	0.90	0.70	10	100.61±1.67	1.66	0.61
	100	98.59±1.22	1.24	-1.41	100	101.28±1.19	1.18	1.28
	150	100.84±1.46	1.45	0.84	150	99.42±0.87	0.88	-0.58
**ATR**	1	100.32±1.03	1.02	0.32	1	99.76±1.40	1.41	-0.24
	20	99.50±1.23	1.24	-0.50	20	100.17±0.69	0.69	0.17
	50	99.65±1.14	1.14	-0.35	50	100.31±1.77	1.76	0.31

[a] Mean ± SD for three determinations.
[b] Percentage relative standard deviation
[c] Percentage relative error.

Table S3: Assay results for the determination of HCT, CPL, LSP, VAL, ATR, VIC, BSL, AML and CVL in their single dosage forms using the proposed MEKC and HPLC methods (n=5)

Dosage form	Proposed MEKC method	Proposed HPLC method	Reference method [c]
**HCT (Laboratory prepared tablets [a])**			
**Mean % recovery ± SD**	101.49±1.70	99.47±0.97	99.2±0.77
RSD%	1.68	0.97	0.78
Er (%)	1.49	-0.53	-0.80
t [b]	2.13	0.37	
F[b]	4.88	1.57	
**CPL (Capoten 50® tablets [a])**			
**Mean % recovery ± SD**	98.77±1.31	--	98.76±0.67
RSD%	1.32	--	0.68
Er (%)	-1.23	--	-1.24
t [b]	0.02	--	
F[b]	3.80	--	
**LSP (Zestril 10® tablets [a])**			
**Mean % recovery ± SD**	--	99.30±1.18	101.08±0.65
RSD%	--	1.19	0.64
Er (%)	--	-0.70	1.08
t [b]	--	2.27	
F[b]	--	3.33	
**VAL (Tareg 80 ® tablets [a])**			
**Mean % recovery ± SD**	98.71±0.79	100.63±1.20	98.62±1.42
RSD%	0.80	1.19	1.44
Er (%)	-1.29	0.63	-1.38
t [b]	0.10	1.87	
F[b]	3.25	5.52	
**ATR (Ator 10 ® tablets [a])**			
**Mean % recovery ± SD**	100.49±0.16	100.08±0.17	100.20±0.11
RSD%	0.16	0.17	0.11

Er (%)	0.48	0.08	0.20
t [b]	2.21	1.03	
F[b]	2.86	2.32	
**VIC (Brain-OX® Capsules [a])**			
**Mean % recovery ± SD**	100.06±0.92	99.89±0.67	98.38±1.02
RSD%	0.92	0.67	1.04
Er (%)	0.06	-0.11	-1.62
t [b]	2.12	2.14	
F[b]	1.24	2.35	
**BSL (Concor 10 ® tablets [a])**			
**Mean % recovery ± SD**	101.77±1.27	99.63±0.60	99.00±1.00
RSD%	1.25	0.61	1.01
Er (%)	1.77	-0.37	-1.00
t [b]	1.86	0.94	
F[b]	3.82	2.74	
**AML (Norvasc 10® tablets [a])**			
**Mean % recovery ± SD**	98.95±1.49	100.10±0.86	99.55±1.69
RSD%	1.51	0.86	1.70
Er (%)	-1.05	0.10	-0.45
t [b]	0.46	0.50	
F[b]	1.28	3.84	
**CVL (Dilatrol 25® tablets [a])**			
**Mean % recovery ± SD**	--	99.59±0.43	99.85±0.62
RSD%	--	0.43	0.62
Er (%)	--	-0.41	-0.15
t [b]	--	0.60	
F[b]	--	2.11	

[a] HCT laboratory made tablets labeled to contain 10 mg HCT/tablet, Capoten 50® tablets labeled to contain 50 mg CPL/tablet, Zestril 10® tablets labeled to contain 10 mg LSP/tablet, Tareg 80® tablets labeled to contain 80 mg VAL/tablet, Ator 10® tablets labeled to contain 10 mg ATR/tablet, Concor 10® tablets labeled to contain 10 mg BSL/tablet, Norvasc 10® tablets labeled to contain 10mg AML/tablet, Dilatrol 25® tablets labeled to contain 25 mg CVL/tablet and Brain Ox® capsules labeled to claim 30 mg VIC/capsule. [b] Theoretical values of t and F are 2.31 and 6.39, respectively, at 95% confidence limit (n=5). [c] Reference methods are: HCT tablets® [1], Capoten 50 ® [2], Zestril 10® [3], Tareg 80® [4], Ator 10® [5], Concor 10® [6], Norvasc 10® [7], Brain OX® [8] and Dilatrol 25® [9].

Table S4: System suitability parameters for the analyzed drugs HCT, CPL, LSP, VAL, ATR, VIC, BSL, AML and CVL using the proposed MEKC and HPLC methods

MEKC method								
**Parameters[a]**	**HCT**	**CPL**	**VAL**	**ATR**	**VIC**	**BSL**	**AML**	
**t_m ± SD (min)**	2.41±0.03	2.91±0.02	3.33±0.05	5.16±0.04	5.85±0.03	6.62±0.02	6.87±0.04	
**Retention factor (k')**	3.60	4.30	4.90	7.80	8.80	9.80	10.20	
**Plate count (N)**	14319	13300	29130	42194	37968	37282	35654	
**Tailing factor (T)**	0.91	1.02	0.89	1.04	1.50	0.82	0.90	
**Selectivity (α)***		1.19	1.14	1.59	1.13	1.11	1.04	
**Resolution (Rs)***		5.49	4.69	14.5	6.25	5.98	2.40	
**HPLC method**								
**Parameters[a]**	**HCT**	**LSP**	**VIC**	**BSL**	**CVL**	**AML**	**VAL**	**ATR**
**t_R ± SD (min)**	3.83±0.03	5.41±0.01	7.16±0.04	8.49±0.04	9.38±0.02	10.37±0.03	10.76±0.02	11.16±0.02
**Retention factor (k')**	2.67	3.92	5.58	6.83	7.58	8.58	8.92	9.33
**Plate count (N)**	3487	8010	29270	49198	87976	98569	119150	128331
**Tailing factor (T)**	0.82	1.27	0.98	0.84	0.85	1.03	0.96	0.92
**Selectivity (α)***		1.47	1.42	1.22	1.11	1.13	1.04	1.05
**Resolution (Rs)***		6.26	8.56	8.26	6.43	2.32	2.95	3.26

[a]The parameters are calculated as per Center for Drug Evaluation and Research, U. S. Food and Drug Administration (U. S. FDA CDER), where k' >2, Rs>2, N>2000 and T≤2

*** The selectivity and Resolution parameters are calculated between the selected peak and the preceded one.**

Table S5: Robustness evaluation for the analysis of HCT, CPL, LSP, VAL, ATR, VIC, BSL, AML and CVL using the proposed MEKC and HPLC methods

MEKC method															
**Parameter**	**Borate buffer concentration, mM (50 ± 2)**			**Buffer pH (9 ± 0.2)**			**Detection wavelength, nm (± 2)**			**Voltage, kV (28,29,30)**			**Sample injection time, s (9, 10,11)**		
	**% Recovery ± SD[a]**	**% RDS[b]**	**tm ± SD[c]**	**% Recovery ± SD**	**% RDS**	**tm ± SD**	**% Recovery ± SD**	**% RDS**	**tm ± SD**	**% Recovery ± SD**	**% RDS**	**tm ± SD**	**% Recovery ± SD**	**% RDS**	**tm ± SD**
**HCT**	100.12±0.34	0.34	2.42±0.01	100.23±0.91	0.91	2.45±0.04	98.09±0.65	0.66	2.43±0.03	99.34±0.43	0.43	2.46±0.02	101.09±0.51	0.51	2.44±0.01
**CPL**	99.67±0.51	0.51	2.90±0.03	101.42±1.23	1.21	2.93±0.02	99.45±0.74	0.74	2.95±0.03	100.81±0.37	0.37	2.94±0.04	100.35±0.29	0.29	2.96±0.01
**VAL**	99.93±0.22	0.22	3.34±0.03	100.06±0.43	0.43	3.31±0.02	98.98±0.51	0.52	3.35±0.01	100.23±0.78	0.78	3.38±0.05	101.01±0.94	0.93	3.34±0.04
**ATR**	99.65±1.07	1.07	5.15±0.03	101.56±0.44	0.43	5.15±0.03	98.57±0.37	0.38	5.15±0.03	99.21±0.96	0.97	5.15±0.03	100.04±0.92	0.92	5.15±0.03
**VIC**	100.83±0.76	0.75	5.82±0.04	100.2±0.66	0.66	5.82±0.04	99.44±0.45	0.45	5.82±0.04	98.97±1.05	1.06	5.82±0.04	100.03±0.99	0.99	5.82±0.04
**BSL**	101.22±0.99	0.98	6.64±0.01	100.32 ±0.89	0.89	6.64±0.01	99.33±0.26	0.26	6.64±0.01	99.56±1.11	1.11	6.64±0.01	100.12±1.03	1.03	6.64±0.01
**AML**	100.13±0.63	0.98	6.86±0.02	99.32±0.33	0.33	6.86±0.02	98.07±0.21	0.21	6.86±0.02	98.93±0.86	0.87	6.86±0.02	100.03±1.00	1.00	6.86±0.02
**HPLC method**															
**Parameter**	**Phosphate buffer concentration, mM (50 ± 2)**			**Buffer pH (3 ± 0.2)**			**Detection wavelength, nm (± 2)**			**Column Temperature °C (24,25,26)**			**Flow rate, mL (0.9, 1, 1.1)**		
	**% Recovery ± SD[a]**	**% RDS[b]**	**t_R ± SD[c]**	**% Recovery ± SD**	**% RDS**	**t_R ± SD**	**% Recovery ± SD**	**% RDS**	**t_R ± SD**	**% Recovery ± SD**	**% RDS**	**t_R ± SD**	**% Recovery ± SD**	**% RDS**	**t_R ± SD**
**HCT**	101.01±1.03	1.02	3.83±0.01	99.95±0.12	0.12	3.83±0.01	101.14±0.99	0.99	3.83±0.01	99.35±0.56	0.57	3.83±0.01	99.19±1.09	1.10	3.83±0.01
**LSP**	99.67±0.32	0.32	5.41±0.03	99.57±0.42	0.42	5.41±0.03	98.84±1.42	1.44	5.41±0.03	99.65±0.44	0.44	5.41±0.03	101.03±1.08	1.07	5.41±0.03
**VIC**	100.24±0.57	0.56	7.16±0.03	99.38±1.19	1.19	7.16±0.03	100.66±0.58	0.58	7.16±0.03	99.88±0.32	0.32	7.16±0.03	99.36±0.62	0.63	7.16±0.03
**BSL**	99.39±0.65	0.66	8.49±0.03	99.47±0.60	0.60	8.49±0.03	100.87±0.76	0.75	8.49±0.03	99.42±0.70	0.70	8.49±0.03	99.18±0.86	0.86	8.49±0.03
**CVL**	100.12±0.11	0.11	9.38±0.04	99.21±0.84	0.85	9.38±0.04	99.45±0.99	0.99	9.38±0.04	99.54±0.94	0.95	9.38±0.04	100.28±1.28	1.28	9.38±0.04
**AML**	99.66±1.16	1.16	10.37±0.01	99.92 ±0.32	0.32	10.37±0.01	100.46±0.42	0.42	10.37±0.01	99.78±0.64	0.65	10.37±0.01	100.62±0.95	0.95	10.37±0.01
**VAL**	100.53±0.48	0.48	10.76±0.02	100.13±0.22	0.22	10.76±0.02	99.29±1.12	1.13	10.76±0.02	99.95±0.21	0.21	10.76±0.02	99.48±0.45	0.45	10.76±0.02
**ATR**	99.66±0.30	0.30	11.16±0.02	101.28±1.11	1.10	11.16±0.02	98.33±1.47	1.48	11.16±0.02	99.37±1.22	1.23	11.16±0.02	100.36±0.31	0.31	11.16±0.02

[a]The mean percentage recoveries for each drug at each parameter at a concentration of 50 µg/mL for HCT, CPL, LSP, ATR, VIC, BSL, AML and CVL and 100 µg/mL for VAL.

[b] RSD percentage of each drug peak area at each studied parameter.

[c] Mean of migration/retention time of each drug±SD.

Table S6: Intra-day and inter-day precision and accuracy for the determination of HCT, CPL, VAL, ATR, VIC, BSL and AML in rat plasma samples using the proposed MEKC method (n = 6)

Precision and Accuracy	Level	Intra-day				Inter-day			
		Nominal Concentration (µg/mL)	Mean % Recovery ± SD	RSD%	Er%	Nominal Concentration (µg/mL)	Mean % Recovery ± SD	RSD%	Er%
HCT	LLOQ	50	105.96±16.67	15.73	5.96	50	103.42±16.67	16.11	3.42
	LQC	200	105.23±14.97	14.22	5.23	200	107.18±8.07	7.53	7.18
	MQC	500	97.39±7.38	7.57	-2.61	500	96.41±5.86	6.08	-3.59
	HQC	1000	100.91±4.32	4.28	0.91	1000	104.09±1.47	1.41	4.09
CPL	LLOQ	100	96.79±15.57	16.09	-3.21	100	104.00±9.28	8.92	4.00
	LQC	300	98.15±5.67	5.78	-1.85	300	99.52±7.81	7.84	-0.48
	MQC	500	91.94±5.94	6.47	-8.06	500	105.76±4.39	4.15	5.76
	HQC	1000	102.47±4.97	4.85	2.47	1000	102.99±5.86	5.69	2.99
VAL	LLOQ	50	105.34±12.45	11.81	5.34	50	92.90±12.45	13.40	-7.10
	LQC	200	104.82±7.36	7.02	4.82	200	103.09±10.22	9.91	3.09
	MQC	1000	98.27±2.18	2.22	-1.73	1000	99.72±2.28	2.28	-0.28
	HQC	2000	100.96±4.15	4.11	0.96	2000	101.65±3.17	3.12	1.65
ATR	LLOQ	50	111.23±8.64	7.77	11.23	50	108.35±8.99	8.30	8.35
	LQC	200	103.78±11.35	10.93	3.78	200	102.34±5.72	5.59	2.34
	MQC	500	94.66±3.07	3.24	-5.34	500	96.39±1.52	1.57	-3.61
	HQC	1000	101.77±6.14	6.04	1.77	1000	99.54±4.17	4.19	-0.46
VIC	LLOQ	10	113.94±13.92	12.22	13.94	10	93.65±11.06	11.81	-6.35
	LQC	50	92.49±10.46	11.31	-7.51	50	100.60±4.43	4.40	0.60
	MQC	500	98.43±3.14	3.19	-1.57	500	97.25±2.03	2.09	-2.75
	HQC	1000	104.83±5.07	4.84	4.83	1000	105.20±4.61	4.38	5.20
BSL	LLOQ	50	97.94±9.29	9.48	-2.06	50	110.33±7.09	6.43	10.33
	LQC	200	92.20±8.71	9.45	-7.80	200	96.07±4.69	4.88	-3.93
	MQC	500	104.98±4.83	4.60	4.98	500	101.27±3.63	3.58	1.27
	HQC	1000	100.63±3.96	3.93	0.63	1000	102.25±2.42	2.36	2.25
AML	LLOQ	50	108.50±11.37	10.48	8.50	50	109.41±7.22	6.60	9.41
	LQC	200	102.66±10.25	9.98	2.66	200	104.03±8.23	7.91	4.03
	MQC	500	96.76±8.20	8.47	-3.24	500	93.12±3.94	4.23	-6.88
	HQC	1000	101.44±2.77	2.73	1.44	1000	100.53±1.40	1.39	0.53

[a] Mean ± SD for six determinations.
[b] Percentage relative standard deviation
[c] Percentage relative error.

Table S7: Intra-day and inter-day precision and accuracy for the determination of HCT, LSP, VIC, BSL, CVL, AML, VAL and ATR in rat plasma samples using the proposed HPLC method (n = 6)

Precision and Accuracy	Intra-day					Inter-day			
	Level	Nominal Concentration (µg/mL)	Mean % Recovery ± SD	RSD%	Er%	Nominal Concentration (µg/mL)	Mean % Recovery ± SD	RSD%	Er%
HCT	LLOQ	1	102.90±16.67	16.20	2.90	1	106.46±11.66	10.95	6.46
	LQC	5	99.04±4.77	4.81	-0.96	5	106.29±8.14	7.66	6.29
	MQC	50	101.76±4.77	4.81	1.76	50	96.89±5.15	5.32	-3.11
	HQC	100	98.92±5.50	5.56	-1.08	100	102.31±2.38	2.33	2.31
LSP	LLOQ	1	102.15±13.62	13.33	2.15	1	99.56±6.27	6.30	-0.44
	LQC	5	103.94±7.27	7.00	3.94	5	98.11±7.40	7.54	-1.89
	MQC	50	96.46±3.86	4.00	-3.54	50	100.43±2.88	2.87	0.43
	HQC	100	105.02±4.02	3.83	5.02	100	101.10±3.48	3.44	1.10
VIC	LLOQ	2	106.65±8.42	7.90	6.65	2	94.72±9.51	10.04	-5.28
	LQC	10	98.86±2.36	2.38	-1.14	10	99.85±4.61	4.62	-0.15
	MQC	50	101.75±7.00	6.88	1.75	50	98.83±3.71	3.75	-1.17
	HQC	100	95.67±4.04	4.22	-4.33	100	100.96±3.07	3.04	0.96
BSL	LLOQ	5	108.12±11.20	10.36	8.12	5	107.48±8.89	8..27	7.48
	LQC	20	98.40±6.41	6.52	-1.60	20	99.32±3.48	3.50	-0.68
	MQC	50	96.71±7.71	7.97	-3.29	50	94.65±6.14	6.48	-5.35
	HQC	100	103.11±1.93	1.88	3.11	100	97.96±3.56	3.63	-2.04
CVL	LLOQ	5	105.90±9.63	9.10	5.90	5	96.48±7.35	7.62	-3.52
	LQC	20	96.33±5.69	5.90	-3.67	20	100.66±2.74	2.73	0.66
	MQC	50	105.33±6.66	6.32	5.33	50	98.14±2.85	2.90	-1.86
	HQC	100	97.67±5.13	5.25	-2.33	100	100.33±7.77	7.74	0.33
AML	LLOQ	1	99.06±7.68	7.75	-0.94	1	108.98±9.32	8.55	8.98
	LQC	5	91.69±7.94	8.66	-8.31	5	94.66±2.59	2.74	-5.34
	MQC	50	103.54±4.18	4.04	3.54	50	102.92±6.48	6.30	2.92
	HQC	100	98.27±1.82	1.85	-1.73	100	98.26±4.60	4.68	-1.74
VAL	LLOQ	2	104.79±5.67	5.41	4.79	2	104.83±6.48	6.18	4.83
	LQC	10	97.79±5.42	5.54	-2.21	10	101.21±6.62	6.54	1.21
	MQC	100	96.53±4.08	4.23	-3.47	100	97.64±2.54	2.60	-2.36
	HQC	200	99.08±2.06	2.08	-0.92	200	100.11±5.14	5.13	0.11
ATR	LLOQ	5	95.69±13.85	14.47	-4.31	5	102.24±6.81	6.66	2.24
	LQC	20	100.34±2.97	2.96	0.34	20	93.06±4.92	5.29	-6.94
	MQC	50	96.78±3.50	3.61	-3.22	50	101.22±4.06	4.01	1.22
	HQC	100	103.44±4.13	3.99	3.44	100	101.76±3.75	3.69	1.76

[a] Mean ± SD for six determinations, [b] Percentage relative standard deviation, [c] Percentage relative error.

Table S8: Stability study for HCT, CPL, VAL, ATR, VIC, BSL and AML using the proposed MEKC method in spiked rat plasma samples (n=6)

Stability condition	Spiked plasma samples	HCT		CPL		VAL		ATR		VIC		BSL		AML	
		Mean %recovery ± SD [a]	RSD% [b]	Mean % recovery ± SD	RSD%	Mean %recovery ± SD	RSD%	Mean %recovery ± SD	RSD%	Mean %recovery ± SD	RSD%	Mean %recovery ± SD	RSD%	Mean %recovery ± SD	RSD%
Freeze and thaw stability	LQC [c]	110.74 ±8.59	7.76	96.53 ±4.01	4.15	95.99 ±2.76	2.88	109.71 ±6.49	5.92	98.66 ±2.26	2.29	92.81 ±4.722	5.09	105.56 ±6.26	5.93
	HQC [d]	92.74 ±7.17	7.74	107.28 ±6.48	6.04	109.88 ±8.75	7.96	97.34 ±2.99	3.08	102.21 ±7.71	7.54	104.53 ±3.34	3.19	92.23 ±3.50	3.80
Short-term temperature stability	LQC	108.03 ±13.11	12.13	105.81 ±3.36	3.17	107.56 ±3.35	3.12	100.62 ±2.61	2.59	90.81 ±7.23	7.96	96.57 ±5.50	5.69	108.38 ±8.01	7.39
	HQC	101.15 ±4.25	4.20	95.34 ±3.75	3.93	91.84 ±9.88	10.76	96.39 ±1.52	1.57	112.07 ±13.99	12.49	106.65 ±5.86	5.49	101.44 ±2.77	2.73
Long-term stability	LQC	103.84 ± 3.91	3.77	104.70 ±4.68	4.47	111.79 ±5.81	5.19	101.20 ±6.46	6.39	96.34 ±2.77	2.87	103.83 ±2.38	2.29	104.733 ±8.14	7.78
	HQC	99.34 ±7.76	7.81	102.28 ±6.42	6.28	94.99 ±2.91	3.07	108.99 ±7.93	7.27	104.00 ±5.53	5.31	105.19 ±5.84	5.55	110.95 ±7.31	6.59
Stock solution stability	LQC	96.59 ±2.20	2.28	98.15 ±5.67	5.78	101.65 ±3.17	3.12	101.61 ±6.25	6.15	100.22 ±5.01	5.00	96.02 ±3.86	4.02	94.05 ±1.79	1.90
	HQC	104.09 ±1.47	1.41	98.00 ±2.94	3.00	105.66 ±5.47	5.18	103.78 ±11.35	10.93	93.44 ±11.07	11.85	91.64 ±7.86	8.58	95.35 ±6.14	6.44

[a] Mean percentage recovery ± standard deviation of six determinations
[b] Relative standard deviation
[c] LQC is 50 µg/mL for VIC, 200 µg/mL for HCT, VAL, ATR, BSL and AML and 300 µg/mL for CPL
[d] HQC is 1000 µg/mL for HCT, CPL, ATR, VIC, BSL and AML and 2000 µg/mL for VAL

Table S9: Stability study for HCT, LSP, VIC, BSL, CVL, AML, VAL and ATR using the proposed HPLC method in spiked rat plasma samples (n=6)

Stability condition	Spiked plasma samples	HCT		LSP		VIC		BSL		CVL		AML		VAL		ATR	
		Mean %recovery ± SD [a]	RSD % [b]	Mean % recovery ± SD [a]	RSD % [b]	Mean %recovery ± SD [a]	RSD % [b]	Mean %recovery ± SD [a]	RSD % [b]	Mean %recovery ± SD [a]	RSD % [b]	Mean %recovery ± SD [a]	RSD % [b]	Mean %recovery ± SD [a]	RSD % [b]	Mean %recovery ± SD [a]	RSD % [b]
Freeze and thaw stability	LQC [c]	100.26 ±2.29	2.29	102.33 ±6.01	5.89	95.70 ±2.70	2.82	104.48 ±4.43	4.24	108.92 ±6.17	5.66	96.93 ±3.00	3.10	95.98 ±5.06	5.27	102.21 ±3.14	3.07
	HQC [d]	100.48 ±2.80	2.79	100.55 ±4.92	4.89	106.59 ±4.07	3.82	92.08 ±2.38	2.58	92.37 ±5.54	6.00	107.83 ±5.64	5.23	105.90 ±5.74	5.42	97.30 ±2.13	2.19
Short-term temperature stability	LQC	99.32 ±0.97	0.98	92.85 ±6.14	6.62	99.19 ±8.50	8.56	106.54 ±4.53	4.25	105.71 ±7.34	6.94	106.17 ±3.79	3.57	102.83 ±1.36	1.33	102.58 ±6.71	6.54
	HQC	97.01 ±1.28	1.32	105.60 ±6.50	6.15	103.56 ±4.45	4.30	102.12 ±3.15	3.08	99.40 ±3.48	3.50	94.12 ±7.76	8.25	95.13 ±4.34	4.56	92.46 ±3.70	4.01
Long-term stability	LQC	101.49 ± 6.17	6.08	98.10 ±1.38	1.41	101.79 ±1.75	1.72	104.04 ±5.66	5.44	103.40 ±3.12	3.02	109.18 ±3.33	3.05	102.27 ±0.51	0.50	108.86 ±3.12	2.85
	HQC	105.34 ±5.56	5.28	102.33 ±3.07	3.00	104.22 ±4.23	4.06	103.29 ±3.73	3.62	101.74 ±3.06	3.01	94.89 ±3.86	4.07	100.67 ±1.56	1.55	107.02 ±6.11	5.71
Stock solution stability	LQC	102.11 ±5.86	5.74	100.93 ±6.22	6.16	94.60 ±2.42	2.55	94.29 ±1.49	1.57	95.37 ±2.11	2.21	101.71 ±3.80	3.74	98.28 ±6.72	6.83	95.78 ±4.51	4.71
	HQC	99.47 ±0.75	0.75	97.67 ±2.31	2.37	98.82 ±6.32	6.40	94.79 ±7.01	7.40	102.58 ±2.17	2.11	105.18 ±4.56	4.34	98.73 ±1.59	1.61	98.54 ±9.68	9.82

[a] Mean percentage recovery ± standard deviation of six determinations
[b] Relative standard deviation
[c] LQC is 5 µg/mL for HCT, LSP, AML, 10 µg/mL for VIC, VAL and 20 µg/mL for BSL, CVL.
[d] HQC is 100 µg/mL for HCT, LSP, VIC, BSL, CVL, AML, ATR and 200 µg/mL for VAL.

Table S10: Greenness assessment of the proposed MEKC and HPLC methods with comparative study with eight reference methods

	Greenness Assessment		
	**Analytical Eco-scale score**	**Green Analytical Procedure Index (GABI)**	**Analytical GREEnness Metric Approach (AGREE)**
**Proposed MEKC method**	**89**	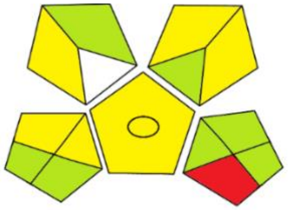	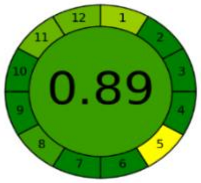
**Proposed HPLC method**	**88**	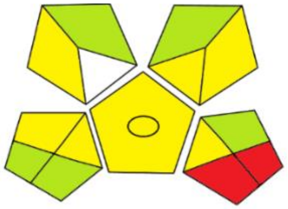	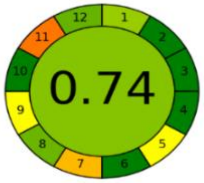
*** Reference 1: HPLC method**	**90**	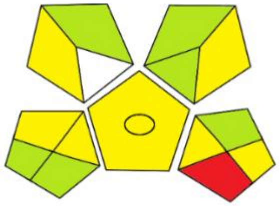	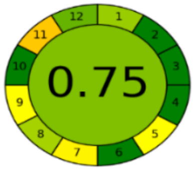

*** Reference 2: Spectrophotometric method**	**91**	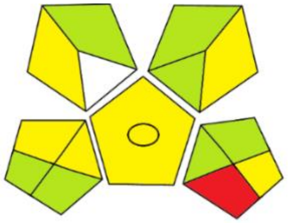	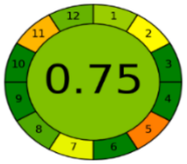

*** The reported reference methods are: Ref. 1 [1], Ref. 2 [10], Ref. 3 [11], Ref. 4 [12], Ref. 5 [13], Ref. 6 [8], Ref.7 [14], Ref. 8 [9].**

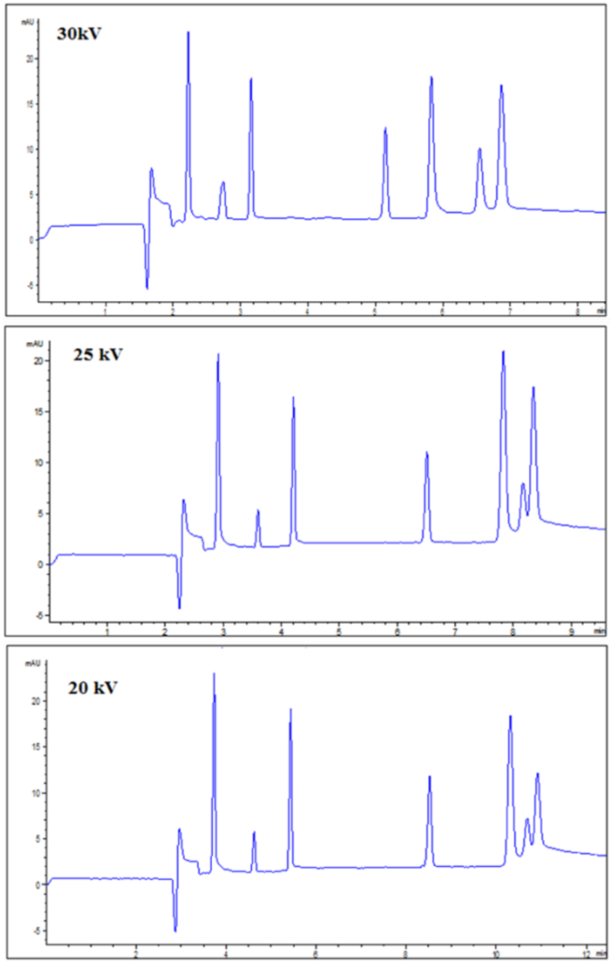

Fig. S1: Effect of the applied voltage on migration times and resolution of the studied drugs.

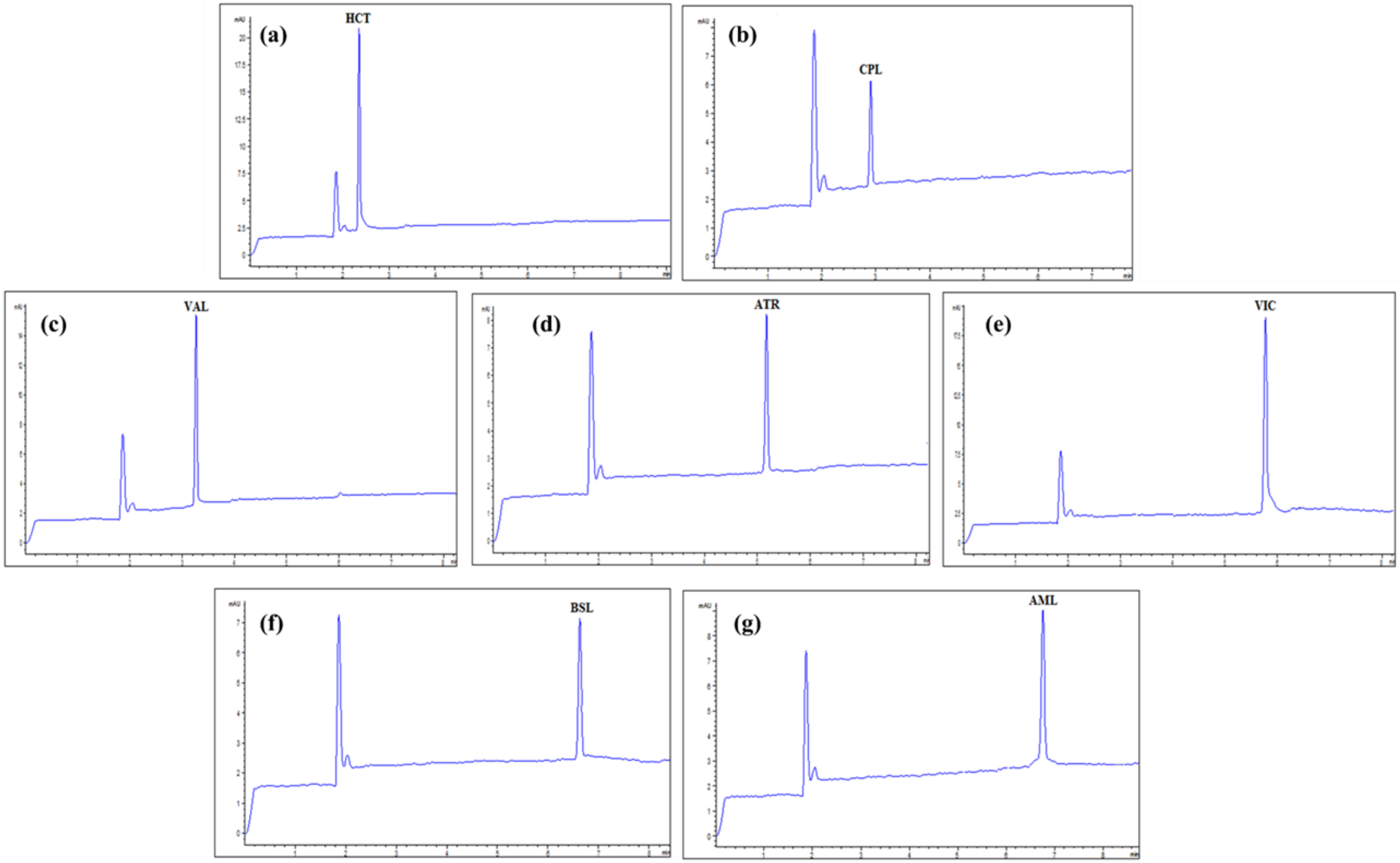

Fig. S2: Electropherograms of 50 µg/mL of HCT, CPL, VAL, ATR, VIC, BSL and AML prepared from single dosage forms; a) HCT laboratory made tablets, b) Capoten 50®, c) Tareg 80®, d) Ator 10®, e) Brain-OX®, f) Concor 10® and g) Norvasc 10® respectively, measured at 220 nm.

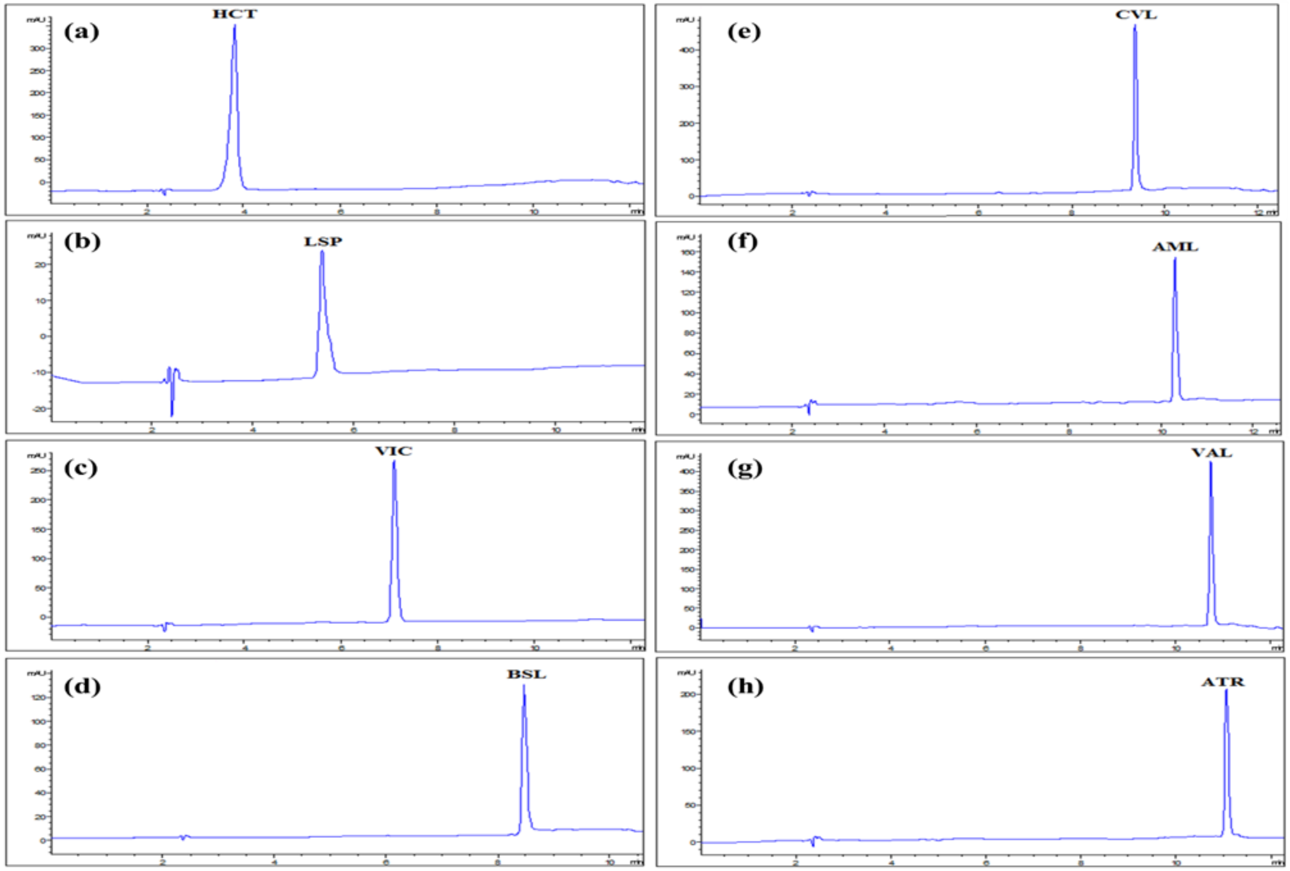

Fig. S3: Chromatograms of 50 µg/mL of HCT, LSP, VIC, BSL, CVL, AML, VAL and ATR prepared from single dosage forms; a) HCT laboratory made tablets; b) Zestril 10®, c) Brain-OX®, d) Concor 10®, e) Dilatrol 25®, f) Norvasc 10®, g) Tareg 80® and h) Ator 10®, respectively, measured at 220 nm.

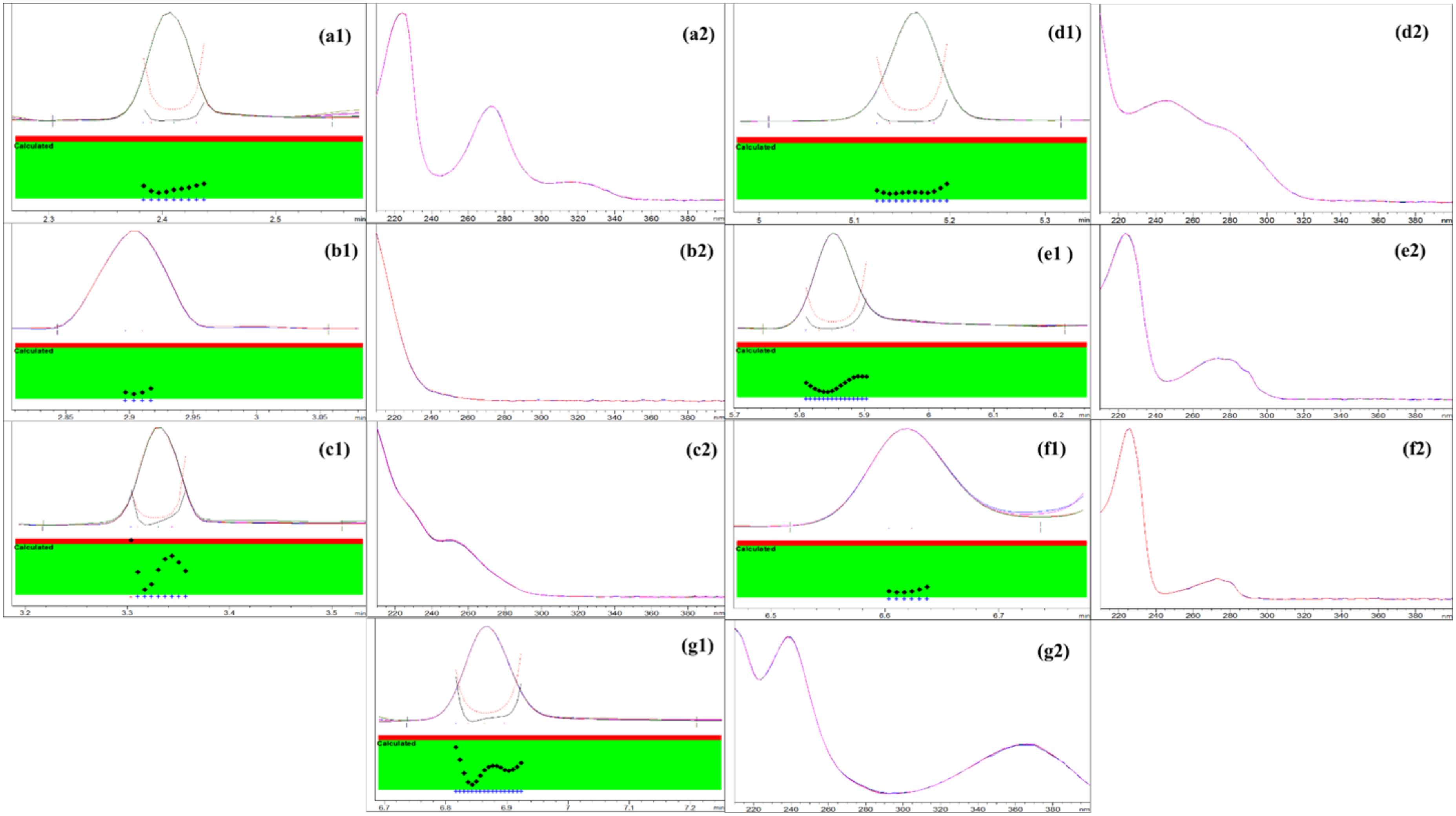

Fig. S4: Peak purity plots (a1-g1) and absorption spectra (a2-g2) measured at 5 different time intervals across the peak for HCT, CPL, VAL, ATR, VIC, BSL and AML, respectively using MEKC proposed method.

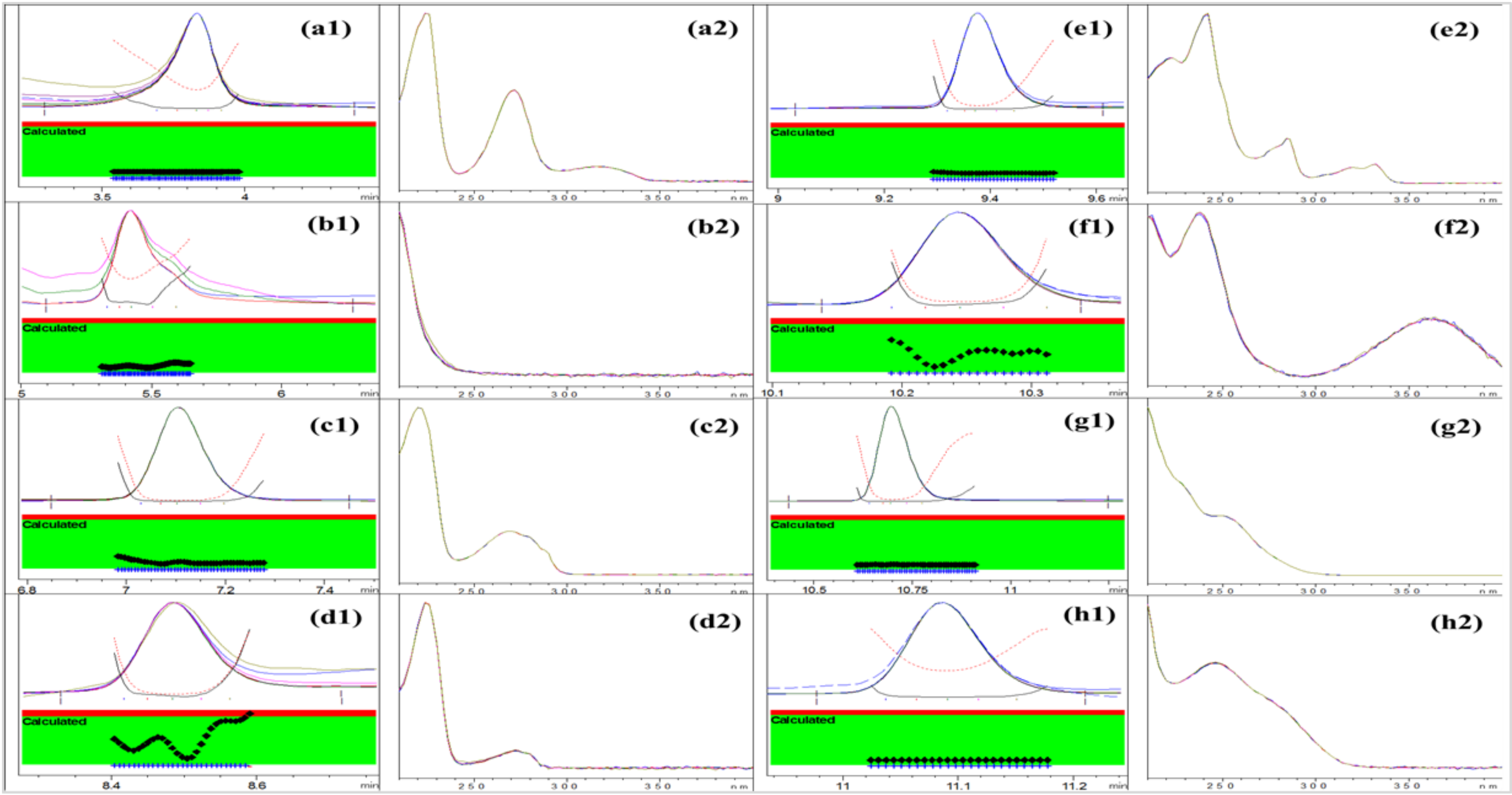

Fig. S5: Peak purity plots (a1-h1) and absorption spectra (a2-h2) measured at 5 different time intervals across the peak for HCT, LSP, VIC, BSL, CVL, AML, VAL and ATR, respectively using HPLC proposed method.

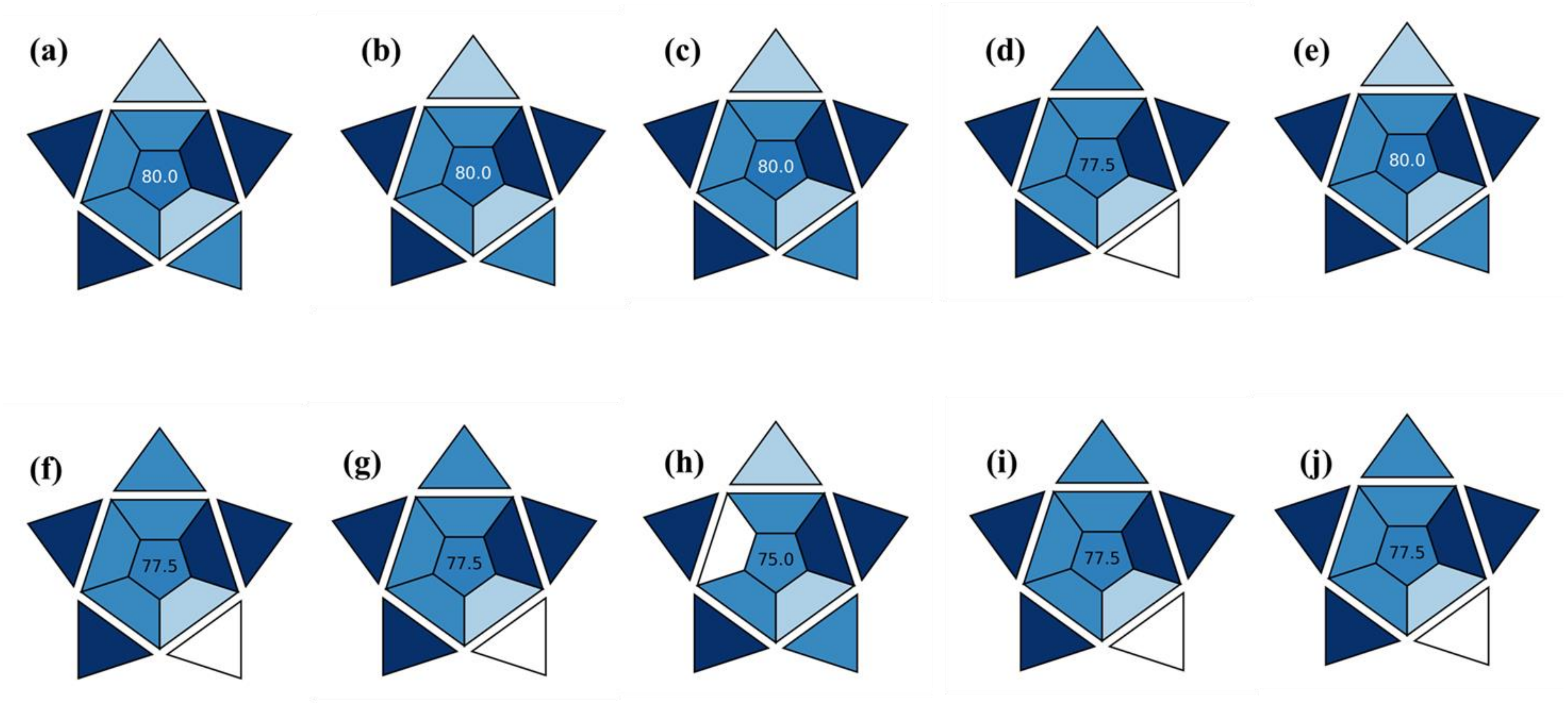

Fig. S6: BAGI index pictograms for ten different analytical methods for the determination of different cardiovascular drugs (a) Proposed MEKC, (b) Proposed HPLC, (c) Reference 1-HPLC, (d) Reference 2-Spectrophotometry, (e) Reference 3- HPLC, (f) Reference 4-Spectrophotometry, (g) Reference 5-Spectrophotometry, (h) Reference 6- HPLC, (i) Reference 7- Spectrophotometry and (j) Reference 8-Spectrophotometry

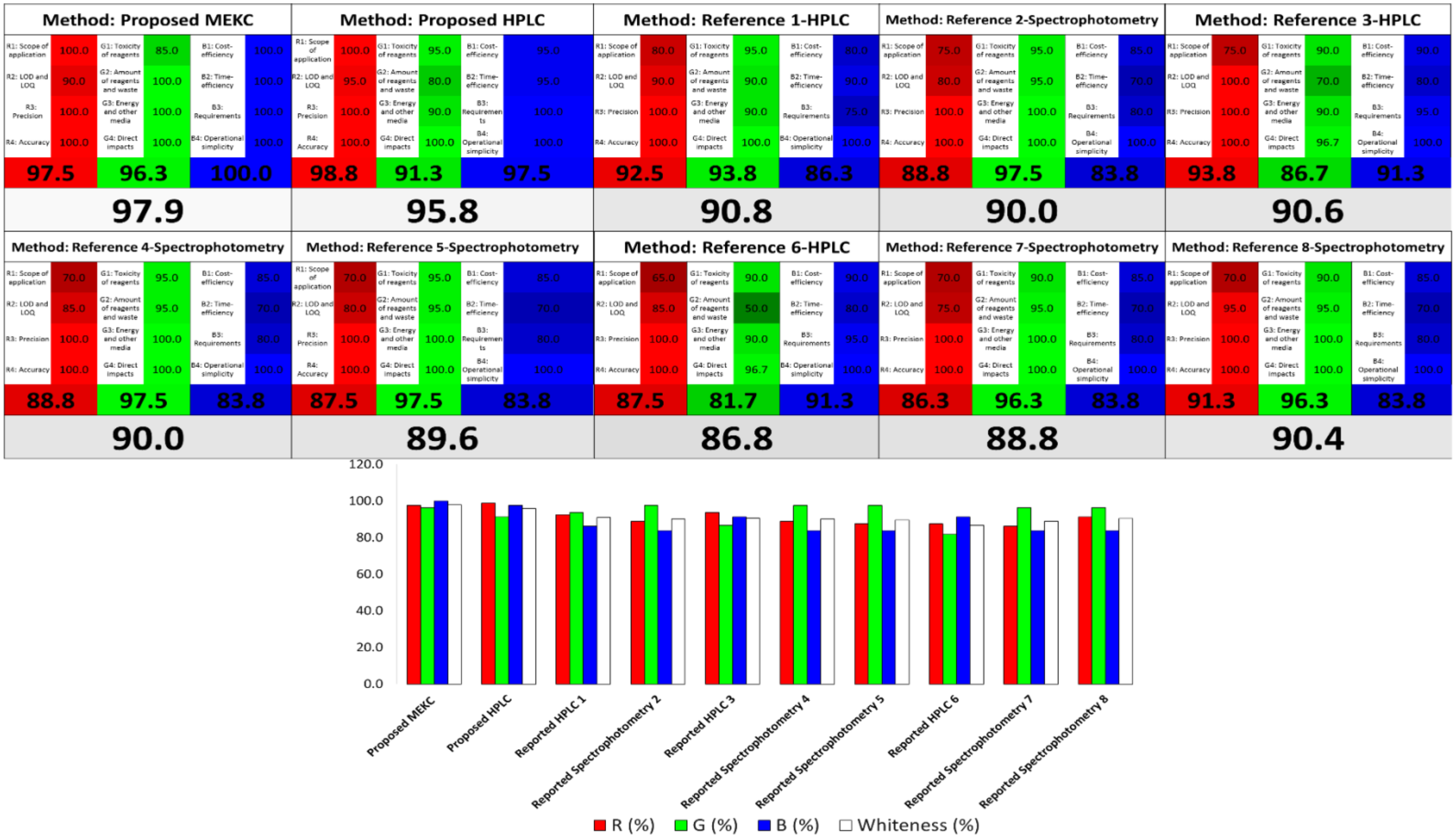

Fig. S7: Comparison of the main evaluation results obtained from RGB12 analysis for the proposed MEKC and HPLC methods with the eight reported methods. The white bar (whiteness %) indicates the arithmetic mean of the three other bars (red, green, and blue)

References:

1. Ahmed, S., N.N. Atia, and N.A. Mohamed, *Dual separation mode for simultaneous determination of antihypertensive drug combinations by high-performance liquid chromatography.* Talanta, 2011. **84**(3): p. 666-672.
2. Chandru, H. and A.C. Sharada, *Simple and Rapid Methods for the Analysis of Captopril in Dosage Forms.* E-Journal of Chemistry, 2007. **4**: p. 915470.
3. Shulyak, N., et al., *Spectrophotometric methods for the determination of lisinopril in medicines.* Pharmacia, 2021. **68**: p. 811-818.
4. Nissankararao, S., et al., *Method development and validation for the estimation of valsartan in bulk and tablet dosage forms by RP-HPLC* Der Pharma Chemica,, 2013. **5**(2): p. 206-211.
5. Ahmad, N., Y. Bitar, and S. Trefi, *Development and validation of a simple method for the determination of Atorvastatin calcium in pure and pharmaceutical formulations using spectrofluorimetry.* Heliyon, 2023. **9**(3): p. e13771.
6. Kondratova, Y., et al., *Development and validation of HPLC-dad method for the determination of bisoprolol in tablet dosage forms.* International Journal of Applied Pharmaceutics, 2017. **9**: p. 54.
7. Mahmoud, A.M., H.M. Abdel-Wadood, and N.A. Mohamed, *Kinetic spectrophotometric method for determination of amlodipine besylate in its pharmaceutical tablets.* J Pharm Anal, 2012. **2**(5): p. 334-341.
8. Shehata, M.A., et al., *Stability-indicating methods for determination of vincamine in presence of its degradation product.* J Pharm Biomed Anal, 2005. **38**(1): p. 72-8.
9. Ansary, A., et al., *Simultaneous Determination of Carvedilol and Hydrochlorothiazide in Tablets and Spiked Human Plasma using Derivative Spectrophotometry.* Pharmaceut Anal Acta, 2012. **3**(9): p. 1-6.
10. Mohamed, N.G., *Simultaneous Determination of Amlodipine and Valsartan.* Analytical Chemistry Insights, 2011. **6**: p. ACI.S7282.
11. Shaalan, R.A., et al., *Validated stability-indicating HPLC-DAD method of analysis for the antihypertensive triple mixture of amlodipine besylate, valsartan and hydrochlorothiazide in their tablets.* Arabian Journal of Chemistry, 2017. **10**: p. S1381-S1394.
12. Elzanfaly, E.S., et al., *Different signal processing techniques of ratio spectra for spectrophotometric resolution of binary mixture of bisoprolol and hydrochlorothiazide; a comparative study.* Spectrochimica Acta Part A: Molecular and Biomolecular Spectroscopy, 2015. **140**: p. 334-343.
13. Magdy, R., et al., *Determination of amlodipine and atorvastatin mixture by different spectrophotometric methods with or without regression equations.* Spectrochimica Acta Part A: Molecular and Biomolecular Spectroscopy, 2019. **210**: p. 203-211.
14. Dinc, S., et al., *Spectrophotometric Multicomponent Resolution of a Tablet Formulation Containing Lisinopril and Hydrochlorothiazide by Multivariate Calibration Methods.* Asian Journal of Chemistry, 2013. **25**(2): p. 999-1002.
